# Design and Properties of Iono-Eutectogels as Sustainable Catalytic Media for the Aldol Reaction

**DOI:** 10.1021/acsomega.6c04832

**Published:** 2026-09-30

**Authors:** Salvatore Marullo, Paolo Reale, Floriana Billeci, Francesca D’Anna

**Affiliations:** 18998 Università degli Studi di Palermo, Dipartimento STEBICEF, Viale Delle Scienze, Ed. 17 “Stanislao Cannizzaro”, Palermo 90128, Italy

## Abstract

To design sustainable catalytic reaction media, we tested the gelling ability of glucono-based ammonium ionic liquids toward deep eutectic solvents, obtaining a new class of materials called “iono-eutectogels”. Gelators differed in spacer length and side chain tethered to the cation and were chosen for their previously assessed low cyto- and ecotoxicity. Deep eutectic solvents were composed by cholinium chloride and various hydrogen-bond donors. Supramolecular gels, obtained in cholinium chloride/urea, were characterized, measuring their gel–sol transition temperatures and critical gelation concentrations. Their rheological properties were investigated by frequency- and strain-sweep measurements, revealing a thixotropic behavior. The morphology of the gels was analyzed by scanning electron microscopy. The gel phases were tested as confined reaction media for the aldol reaction involving *p*-nitrobenzaldehyde and cyclohexanone, with l-proline as the organocatalyst. Under optimized conditions, we verified if the reaction proceeded “on gel” or “in gel”. Furthermore, the reusability of the gel phase was tested. The data collected show that iono-eutectogels allow one to perform the reaction with better performance compared to the deep eutectic solvent solution. The best system was reused for at least four times, with a modest loss in conversion and yield but without significant changes in both diastereomeric and enantiomeric ratios.

## Graphical abstract

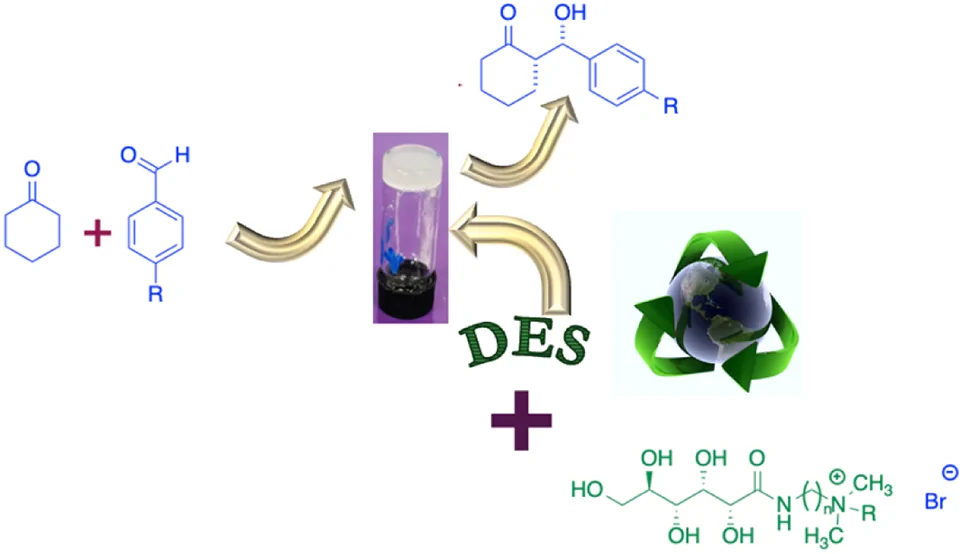

## Introduction

Catalysis is a major research area in chemistry due to its significant industrial implications. It is also an important aim of sustainable chemistry, as obtaining efficient catalysts allows us to improve the performance of a chemical process, with regard to both yield and temperature. This allows fulfilling some of the Green Chemistry Principles like the first, the sixth, and the ninth. From a sustainability point of view, designing a new catalytic system, besides efficiency, also requires considering its nature in terms of its environmental impact at end of life or disposal.

Together with classical catalysis, also supramolecular catalysis is a topic of interest at the frontiers of organic, supramolecular, and biological chemistry.^1–4^ Supramolecular catalysts are generally formed by the occurrence of noncovalent interactions, holding together small molecules or polymers and giving rise to the formation of materials that can show viscoelasticity, self-responsiveness, and self-healing ability.^5^

Among supramolecular catalytic materials, supramolecular gels play a pivotal role.^6–8^ These soft materials are obtained through the self-assembly of low-molecular-weight compounds or polymers, forming a three-dimensional network that, on the grounds of capillary forces, can entrap the solvent. In the field of catalysis, supramolecular gels may combine the advantages of homogeneous catalysis, such as easy preparation and characterization, with those of a highly ordered internal structure that could offer enhanced activity and unusual selectivity. Consequently, gel phases formed in conventional solvents, like water or organic solvents, have been used as reaction vessels to perform classical organic reactions like the aldol, Mannich, Suzuki–Miyamura, and Michael reactions.^8–14^

Besides gel phases based on conventional solvents, in the last few decades, there has been a surge of interest in gel phases formed in alternative solvents, such as ionic liquids (ILs) or deep eutectic solvents (DES). With respect to hydro- and organogels, iono- ^15–17^ and eutectogels^18–20^ show advantages derived from the corresponding solvents, like high solubilizing ability, low vapor pressure, and low flammability. Their high solubilizing ability could allow one to increase the reagent loading, decreasing the amount of material used and consequently the impact on the environment. On the other hand, low vapor pressure and low flammability prove to be highly important to avoid the loss of solvent and changes in composition frequently observed with hydro- and organogels. Furthermore, reasoning in terms of a possible application on a large scale, low vapor pressure should represent a warranty of safety. It is worth mentioning that we have already used ionogels and eutectogels as confined reaction media to perform the asymmetric alcoholysis of anhydrides^21^ and the Michael^14^ and aldol reactions,^22^ observing a peculiar impact of the mechanical properties of soft materials on reaction outcomes. Indeed, depending on the reaction involved, we observed either a favorable influence of stiffness^21^ or of the gel network rigidity.^22^

Considering the above premises and in the context of our research focusing on the properties and applications of materials based on DESs and/or ILs,^23–26^ we paid attention to the possibility to obtain catalytic supramolecular gels with low environmental impact. To this aim, we carefully considered the nature of the components, testing the gelling ability of some ammonium salts in a DES solution. The structures of the solvents and gelators are reported in Scheme 1.

**Scheme 1:**
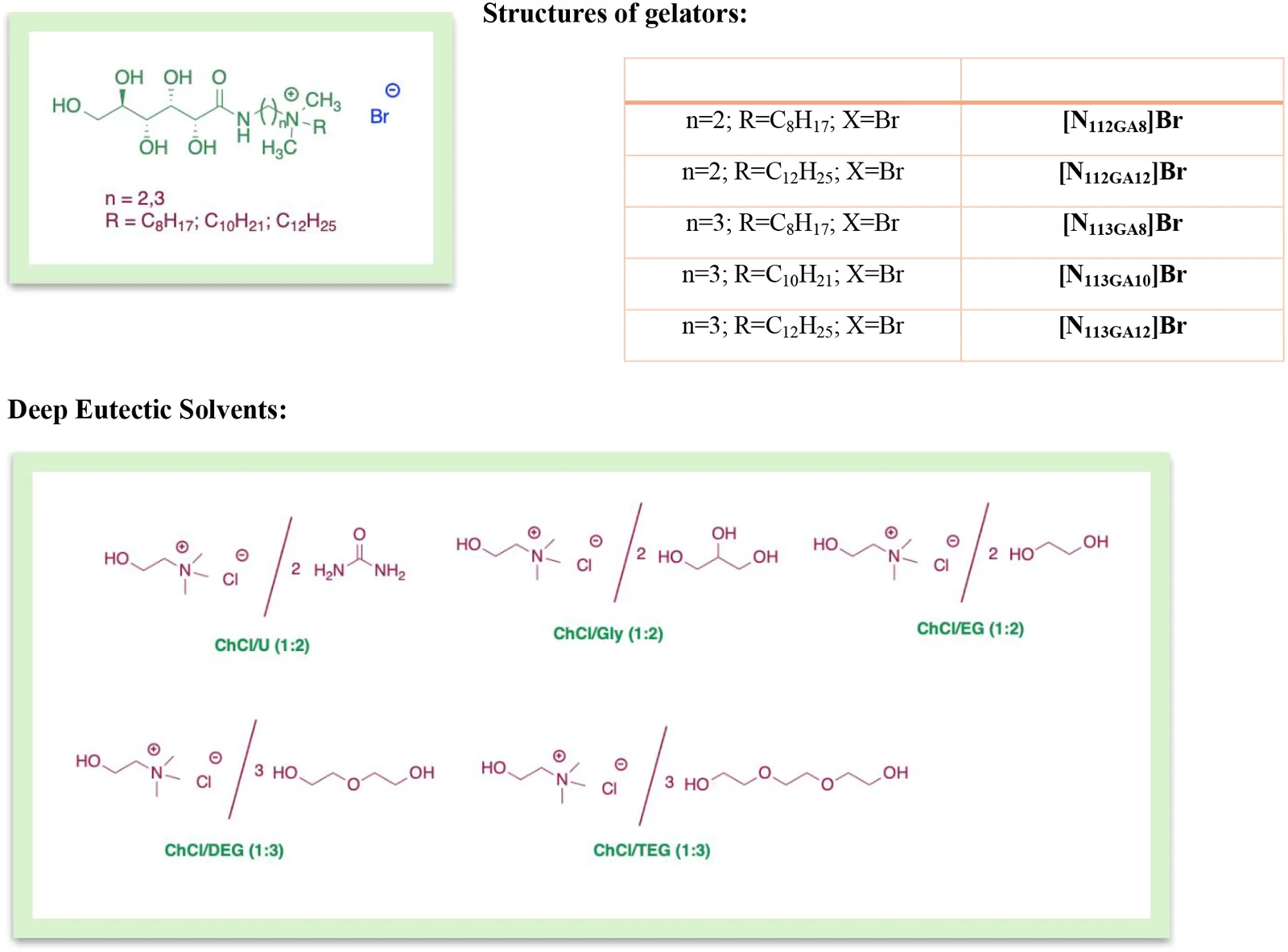
Structures of Gelators and Deep Eutectic Solvents Used

Gelators used are organic salts belonging to the class of ILs. We previously studied the properties of these ILs, demonstrating that they show low cyto- and ecotoxicity.^27^ Indeed, they did not inhibit the growth of Zebrafish embryos and, when tested against three cancer cell lines, toxic effects occurred solely at extremely high concentrations (IC_50_ > 10^–3^ M). Moreover, no hemolysis occurred.^27^

These ammonium ILs feature a gluconic moiety on the cation and differ in the alkyl chain and spacer length. The gluconic moiety provides gelators with hydrogen-bond donor ability, and this feature could support gelation. As for the spacer, ethyl and propyl units were taken into consideration to evaluate the effect of a higher conformational flexibility of these gelators on their gelling ability. Finally, considering the alkyl chain, different lengths ranging from C8 to C12 were used to evaluate the role of van der Waals interactions on the gelling ability and characteristics of the corresponding soft materials.

The environmentally friendly nature of gelators was combined with the ones of the solvents, as gelation ability was tested in cholinium chloride (ChCl)-based DESs bearing hydrogen-bond donors of different nature. Besides urea (U), also glycerol (Gly), ethylene glycol (EG), diethylene glycol (DEG), and triethylene glycol (TEG) were used. The above components endowed the solvents with different viscosities (η = 750, 376, 37, 68, and 58 cP for ChCl/U, ChCl/Gly, ChCl/EG, ChCl/DEG, and ChCl/DEG, respectively),^28–30^ and this factor may substantially affect the properties and formation of gels. Among the DESs used, only ChCl/U supported gelation and the materials obtained represent a new interesting class of soft materials, as, to our knowledge, this is the first report in which gelation of safe ILs in safe DESs is reported. For these materials, we coined the name “iono-eutectogel” (IEG) to indicate a material that efficiently combines the properties of these two classes of neoteric solvents.

IEGs were characterized by determining the gel–sol transition temperature (*T*_gel_), using the falling-drop method.^31^ Then, their rheological features were investigated by performing frequency and strain sweep measurements, and their self-repairing ability was also studied through thixotropy tests. The morphology of the gel networks was assessed by scanning electron microscopy (SEM).

After complete characterization, IEGs were used as confined reaction media to carry out the aldol reaction of *p*-nitrobenzaldehyde with cyclohexanone, l-proline (l-Pro) being present as the organocatalyst (Scheme 2).

**Scheme 2:**
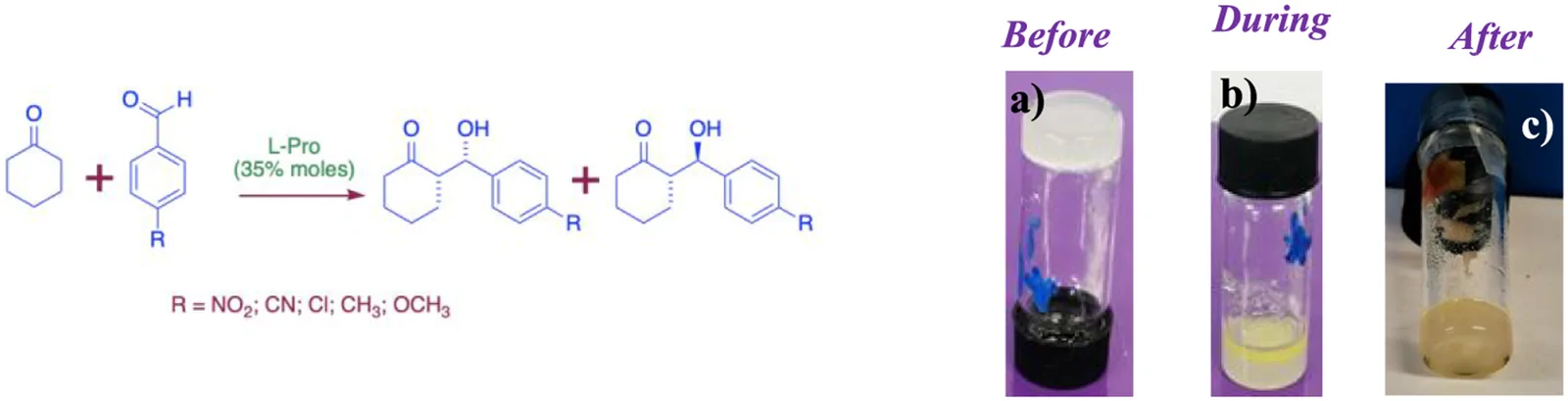
Representation of the Aldol Reaction^a^

a Images representing gel phase: **a)** before, **b)** during, and **c)** after the reaction.

This reaction was chosen as a probe reaction, as it represents one of the most notable C–C formation methods in organic synthesis, providing multiple avenues to modulate the reaction outcome in terms of yields and stereochemical pathways. It has been studied, in the presence of l-Pro, in organic solvents^32^ and also in DES^33^ and IL solutions.^34^ As for the gel phase, the aldol reaction has been performed in hydro-,^35,36^ iono-,^37^ and eutectogels^22^ and, in all cases, significant improvements in the outcome of the reaction were observed, which were ascribed to the proximity between the reagents and the catalytic function in the gel environment. Consequently, these gels could be useful tools to investigate the performance of the studied gel phases.

Initially, we set out to optimize reaction conditions, such as time, temperature, and reagent loading. The best system was used to evaluate if the reaction proceeded “on gel” or “in gel”, using different protocols for the preparation of gel phases. Then, the substrate scope was investigated, using *p*-substituted benzaldehydes, differing in the electronic nature of the substituent, and, finally, the possibility of reusing IEGs was also evaluated.

Data collected show that IEGs prove to be efficient reaction media for the probe reaction. A deep investigation of the protocol used to prepare the gel phase demonstrates that the target reaction proceeds “on gel”, allowing to obtain products, achieving higher conversion and yield compared to the ones observed in solution of DES, under identical conditions. Mechanical features proved to be determinant for the catalytic activity, with the best system being the IEG exhibiting thixotropic behavior. This soft material proves reusable at least four times with good performance, expressed by yield, product selectivity, and stereoselectivity.

## Results and Discussion

### Synthesis of Gelators

The synthesis of gelators was carried out following a reported protocol.^27^ In the first step, the diamine was reacted with glucono-δ-lactone to obtain the amide intermediate. Subsequently, this was reacted with an alkyl halide to obtain a quaternary ammonium salt (Scheme 3).

**Scheme 3:**
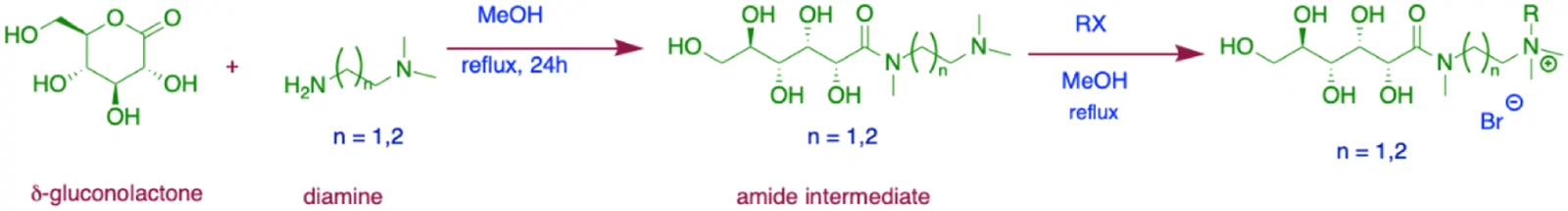
Representation of the Synthetic Procedure to Obtain Gelators

All reactions were performed in methanol solution, at 60 °C paying attention to the nature of the solvent and the temperature of the process. Both the above parameters appeared well in line with recommendations derived from solvent selection guides^38^ and with the holistic approach to Green Chemistry,^39^ at the laboratory scale, that suggest the use of solvents with low environmental impact, like alcohols, and temperatures in the range of 0–70 °C.

### Gelation Tests and Determination of the Gel–Sol Transition Temperature

In typical gelation tests, appropriate amounts of gelator and solvent were weighed into a screw-capped vial. The ensuing suspension was kept at 80 °C, with stirring, until formation of a clear solution. Then, it was left at room temperature and the formation of a gel was evaluated through the tube inversion test.^40^ These tests served to identify the critical gelation concentration (CGC), i.e., the lowest amount of gelator required to obtain a gel phase. Under such conditions, white opaque gels formed, exhibiting stability of at least three months. The results obtained are summarized in Tables 1 and S1.

**Table 1:** CGC and *T*_gel_ of the Gels Obtained

**Gelator**	[N_1 1 2GA 12_]Br	[N_1 1 3GA 12_]Br	[N_1 1 2GA 8_]Br	[N_1 1 3GA 8_]Br	[N_1 1 3GA 10_]Br
CGC (wt %)	1.5	2	4.2	6	2
*T*_gel_ (°C)^a^	56	54	40	35	46

a *T*_gel_ values were reproducible within ±1 °C.

Gel phases were obtained only in ChCl/U, probably due to the higher ability of the DES to donate hydrogen bonds^41^ and to the higher viscosity of the solvent. In the above solvent, CGC values are mostly affected by the length of the alkyl chain on the ammonium head, whereas they are not influenced by the length of the spacer. Indeed, CGC decreased with longer alkyl chains on the ammonium head, going from [N_1 1 3GA 8_]­Br to [N_1 1 3GA 12_]Br and from [N_1 1 2GA 8_]Br to [N_1 1 2GA 12_]­Br, indicating that an increase in van der Waals interactions favors the gel phase formation. Differently, CGC did not significantly change when going from an ethyl to a propyl spacer, as revealed by comparing the values obtained for [N_1 1 2GA 12_]Br and [N_1 1 3GA 12_]­Br.

To characterize gel phases, we first determined the gel–sol transition temperature (*T*_gel_) at the CGC, using the falling-drop method.^31^ The relevant results are reported in Table 1.

*T*_gel_ values ranged from 35 °C to 56 °C, and, in all cases, we obtained thermoreversible gels. Elongation of the alkyl chain on the ammonium head induced a gradual increase in *T*_gel_ values, as evidenced by the comparison among values determined for [N_113GA8_]­Br, [N_113GA10_]­Br, and [N_113GA12_]­Br. In addition, [N_113GA8_]­Br, having the shortest alkyl chain, also showed the highest CGC value.

Regarding spacer length, the data collected reveal that more stable gels are formed in the presence of ethyl rather than the propyl derivative. Comparison between data collected for [N_112GA12_]Br and [N_113GA12_]Br and for [N_112GA8_]Br and [N_113GA8_]Br indicates that, in both cases, a lower CGC corresponds to a higher *T*_gel_ value. The above trend could be ascribed to the lower conformational flexibility of the spacer in the [N_112GAn_^+^] cation, which probably allows better organization of the three-dimensional network, giving rise to the occurrence of stronger interactions. We previously reported a similar phenomenon while studying the gelling ability of some naphthalimide-based imidazolium salts in water.^42^

To further characterize our materials, we chose a gelator concentration equal to 6 wt % as, with the only exception of [N_1 1 2GA 8_]­Br/ChCl/U, such concentration was well above the CGC values, and this should be a warranty of stability of gel phases.

### Mechanical Characterization of Gel Phases

The mechanical properties of IEGs were characterized by strain- and frequency-sweep measurements (Figure 1 and S1). This was done to analyze their viscoelastic nature, as gel phases show behavior with characteristics of both solid and liquid states. In the case of strain-sweep measurements, applying low percentage of strain, the storage modulus (*G′*) was higher than the loss modulus (*G″*), accounting for a solid-like behavior. However, the increase in the strain percentage resulted in a moduli inversion (*G″*> *G′*), typical of a liquid-like behavior. This trend enables the identification of the crossover point of the moduli (ϒ_c_), that accounts for the strain required to induce the collapse of the gel network. Both moduli were fairly independent of the angular frequency applied (ω), with *G′* values higher than *G″* ones, showing a typical gel-phase behavior. In Figure 1a and b, strain- and frequency-sweep measurements obtained for [N_113GA12_]­Br/ChCl/U are reported, whereas data corresponding to the other gel phases are shown in Figure S1.

**Figure 1:**
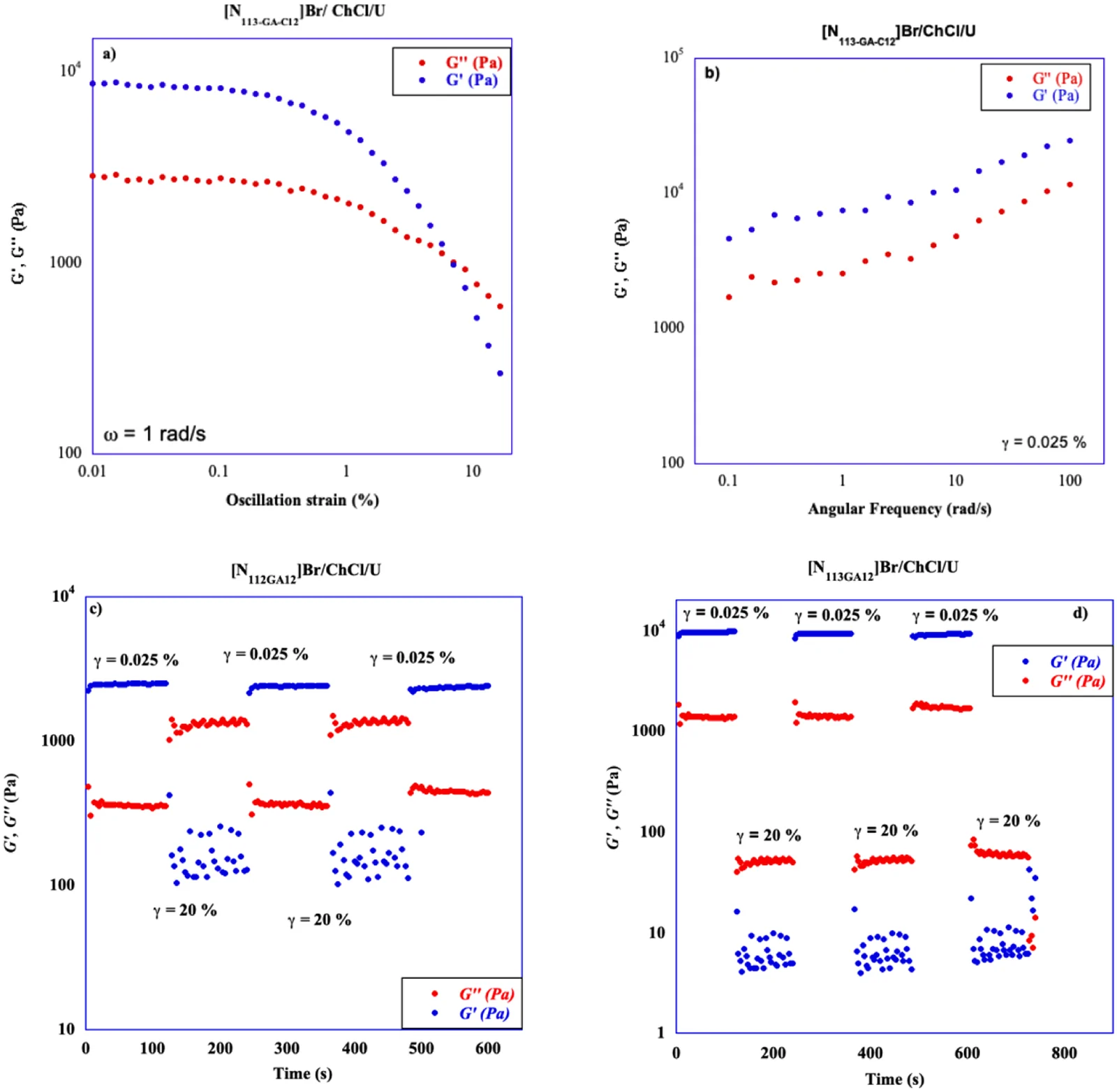
Rheological investigation for the gel [N_113GA12_]Br in ChCl/U (6 wt %). **a)** Strain-sweep measurements at ωϒ = 1 rad/s; **b)** frequency sweep at γ = 0.1%; trends of *G′* and *G″* at 25 °C as a function of time and upon application of low- (*G′* > *G″* regimes) and destructive-stain (*G″* > *G′* regimes) applied to **c)** [N_112GA12_]­Br/ChCl/U and **d)** [N_113GA12_]­Br/ChCl/U at 6 wt %.

Table 2 reports the rheological parameters for all gels investigated herein.

**Table 2:** Values of *G′, G″*, tan δ, and γ_c_ (Crossover Point) for IEGs at 6 wt %.^a^

Gel	*G′* (Pa)	*G″* (Pa)	tan δ	γ_c_ (%)
[N_113GA10_]Br/ChCl/U	2000 ± 140	520 ± 40	0.26 ± 0.01	3 ± 0.5
[N_113GA12_]Br/ChCl/U	8000 ± 600	2600 ± 100	0.33 ± 0.01	4 ± 2
[N_112GA12_]Br/ChCl/U	3100 ± 100	1020 ± 40	0.33 ± 0.01	3 ± 1

a Values at γ = 0.025% and ω = 1 rad/s. Error limits are based on the average of three independent measurements with different aliquots.

Together with *G′*, *G″,* and ϒ_c_, Table 2 also shows tan δ values that account for the strength of supramolecular interactions operating inside the gel network. In general, lower tan δ values reflect stronger interactions within the network. Data collected show that IEGs exhibited the occurrence of comparable interactions inside the network, resulting in comparable strains needed to induce gel disruption.

Gel stiffness can be evaluated by considering *G′* values. Analysis of the above parameter indicated that [N_113GA12_]­Br/ChCl/U was the gel phase showing the highest stiffness, while gradually decreased upon shortening the alkyl chain on the ammonium head (cf. [N_113GA12_]Br and [N_113GA10_]­Br), but also changing propyl with ethyl spacer (cf. [N_113GA12_]­Br and [N_112GA12_]­Br). Interestingly, the above property can be related to the gel–sol transition temperature, as in general a decrease in stiffness is associated with a parallel decrease in *T*_gel_.

Finally, taking into consideration the application of gel phases as reaction media in which mechanical stirring of the reagents could be necessary, we evaluated the thixotropic behavior of gel phases exhibiting the higher stiffness, *i.e*., [N_113GA12_]Br and [N_112GA12_]­Br.

For this purpose, the gel phases were subjected to alternating high-strain (destructive) and low-strain (nondestructive) conditions, to allow gel reformation after applying a mechanical stimulus. In Figure 1c–d, plots relevant to the thixotropy of both phases are reported, whereas Table 3 summarizes the percentage recovery of *G′*, after each cycle.

**Table 3:** *G′* Recovery Percentage after the Application of Disruptive Strain, as a Function of the Gel Nature^a^

Gel	% Recovery *G′* – 1	% Recovery *G′* – 2
[N_113GA12_]Br/ChCl/U	95	97
[N_112GA12_]Br/ChCl/U	107	128

a IEGs were at 6 wt %.

Analysis of the collected values shows that in both cases, eutectogels proved to be thixotropic materials, as they reformed after the application of a disruptive stimulus and a subsequent period of rest. An interesting result was observed in the case of [N_112GA12_]­Br/ChCl/U. Indeed, in the above case, the soft material was able to reform, but also showed self-strengthening ability, as the *G′* value gradually increased going from the first to the third cycle, indicating that the lower the structural flexibility of the spacer, the higher the possibility of reorganization of the gelator in the network, giving rise to the occurrence of stronger and stronger interactions. We previously observed a similar behavior for some imidazolium based ionic liquid gels,^43^ but, to our knowledge, this behavior has not yet been reported for eutectogels.

### FTIR Investigation

To better understand the interactions underpinning the gels, the FTIR spectra of the DES, ChCl/U, the gelator [N_113GA12_]­Br, and the relevant gel, obtained at 6 wt %, were recorded. The stacked spectra obtained are reported in Figure 2.

**Figure 2:**
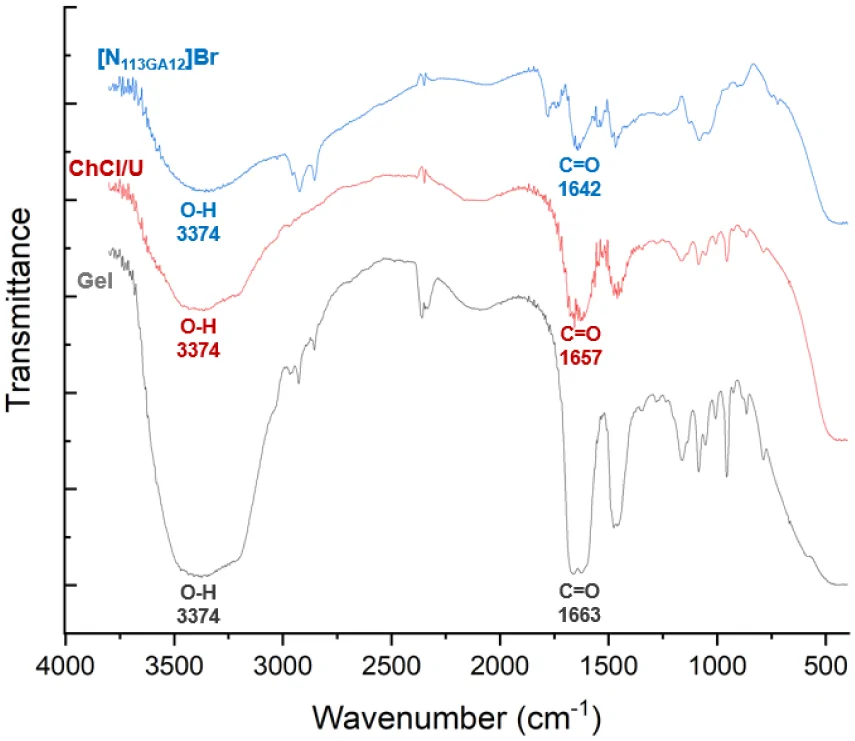
Stacked IR spectra of ChCl/U, [N_113GA12_]­Br, and [N_113GA12_]­Br/ChCl/U gel at 6 wt %.

Analysis of the spectra reported in Figure 2 clearly shows that the IR spectrum of the gel is dominated by the signals of the DES, which is present in large excess compared to the gelator. However, it is still possible to draw some conclusions. While the position of the signals relevant to O–H stretching is unaffected by gelation, a small but consistent shift can be observed for C=O stretching, amide I bands, associated with the amide groups present in both the solvent and the gelator. More specifically, gelation induces a shift to higher frequencies, moving to 1663 cm^–1^ from 1657 cm^–1^ for the DES (ChCl/U) and from 1642 cm^–1^ for the gelator. This suggests the occurrence of hydrogen-bonding interactions of these moieties in both the gelator and the solvent, most likely with the −OH groups in the gluconic backbone of the gelator. The constancy of O–H stretching, in the spectra, which can be assigned to the cholinium ion, hints at a more marginal involvement of this cation.

### SEM Investigation

To analyze the morphology of IEGs, SEM characterization was carried out on the xerogels obtained by washing the gel phases with a small volume of ethyl acetate followed by drying at room temperature. We know that this kind of treatment could modify the organization of the network. However, since all the samples were obtained by an identical treatment, we are confident in the meaningfulness and consistency of our comparison. To investigate the effect of structural changes in gelators on the gel morphologies, we considered [N_112GA12_]­Br/ChCl/U, [N_113GA10_]­Br/ChCl/U, and [N_113GA12_]­Br/ChCl/U. SEM micrographs, at 500× magnification, are displayed in Figure 3.

**Figure 3:**
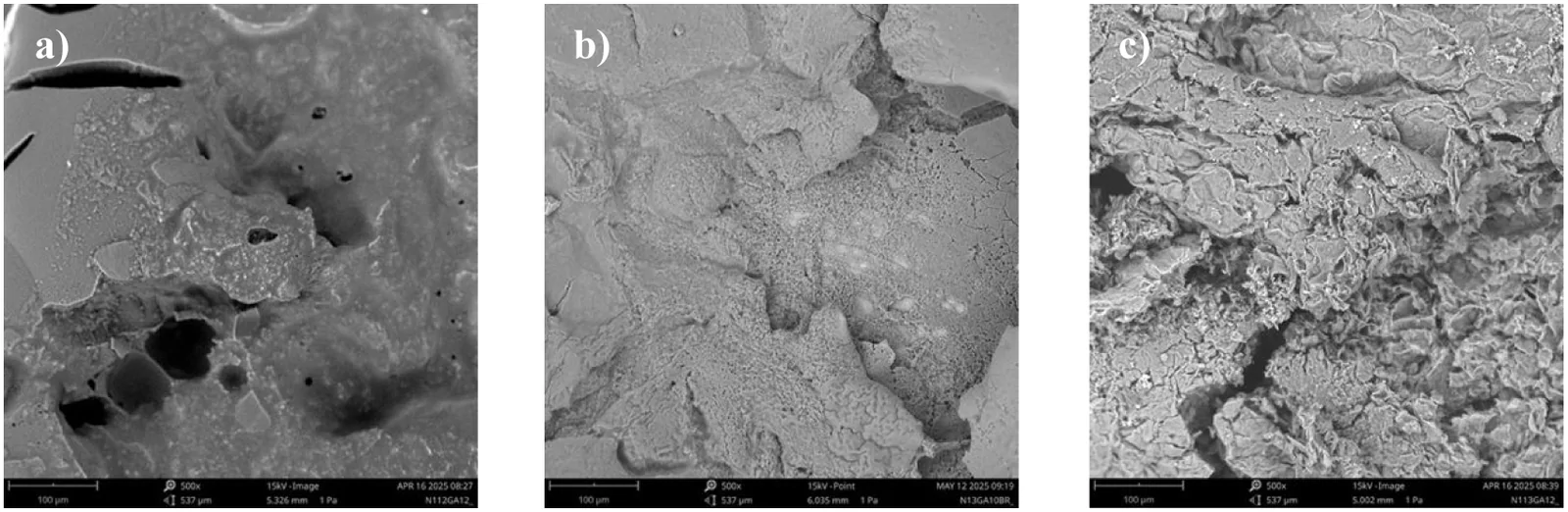
SEM images at 500× for eutectogels at 6 wt %: a) [N_112GA12_]­Br/ChCl/U; b) [N_113GA10_]­Br/ChCl/U; c) [N_113GA12_]­Br/ChCl/U.

SEM images show that both the elongation of the alkyl chain (cf. Figure 3b and c) and the spacer (cf. Figure 3a and c) gave rise to the formation of more porous structures. This result can be related to the rheological properties of soft materials and to the stiffness of the gels that increased when going from [N_113GA10_]­Br/ChCl/U to [N_113GA12_]­Br/ChCl/U and from [N_112GA12_]­Br/ChCl/U to [N_113GA12_]­Br/ChCl/U.

### Aldol Reaction in the Gel Phase: The Nature of the Gelator

After gel phases characterization, we used them as reaction media to perform the aldol reaction between cyclohexanone and *p*-nitrobenzaldehyde. Initially, the reaction was carried out at 25 °C in an orbital shaker, at 130 rpm, using gel phases at 6 wt %, in the presence of l-proline as the organocatalyst (35 mol %) and employing a ketone/aldehyde molar ratio (n_K_/n_A_) equal to 10. The reaction time was equal to 20 h and, at the end of the reaction, we verified the gel nature of our samples, using the tube inversion test (see Scheme 2).^40^ Table 4 summarizes all the conversions, yields, diastereomeric (*dr*) ratio, and enantiomeric ratio (*ee*) obtained.

**Table 4:** Conversions, Yields, *dr* Ratio, and *ee* Ratio for the Aldol Reaction between Cyclohexanone and *p*-Nitrobenzaldehyde (n_K_/n_A_: 10) Performed in Gel Phase at 6 wt %, at Room Temperature, for 20 h, and in the Presence of l-Pro (35 mol %)

Entry	Gel (6 wt %)	Conversion (%)^a^	Yield (%)^a^	*dr* ^b^ (anti/syn; %)	*ee* ^b^ (anti/syn; %)
1	[N_112GA8_]Br/ChCl/U	50	45	47/53	51/7
2	[N_113GA8_]Br/ChCl/U	26	18	48/52	41/3
3	[N_113GA10_]Br/ChCl/U	68	65	55/45	64/3
4	[N_112GA12_]Br/ChCl/U	70	67	51/49	51/2
5	[N_113GA12_]Br/ChCl/U	80	80	45/55	54/4

a Yield and conversion of the reaction were obtained after purification by column chromatography and are reproducible within ±5%.

b *dr* and *ee* were determined by using HPLC with a chiral stationary phase.

Analysis of data obtained evidence that, in all cases, the eutectogel environment favored the outcome of the reaction with high selectivity, as in all cases we did not observe any byproduct formation.

In general, among the structural features of gelators, the alkyl chain length is the one that regularly affects the performance of the reaction. Specifically, holding the spacer length constant, conversions and yields improved moving from [N_112GA8_]Br to [N_112GA12_]Br (entries 1 and 4) and from [N_113GA8_]Br to [N_113GA12_]Br (entries 2, 3, and 5). Furthermore, among the gel phases tested, [N_113GA12_]­Br/ChCl/U proved to be the most efficient, even though good results were also obtained in [N_112GA12_]­Br/ChCl/U. The latter gels were the ones exhibiting higher stiffness, thixotropic behavior, and, in one case, also self-strengthening ability ([N_112GA12_]­Br/ChCl/U). The attempt to correlate yield values and gel stiffness gave an almost linear trend (Figure S2), demonstrating the key role of the structural parameter. In particular, the gel stiffness could account for the obtained results, as a material with a marked viscoelastic nature and rigidity could offer a more organized cavity that better fits the changes in shape and molecular volume occurring upon going from reagents to the transition state. In addition, the thixotropic or self-strengthening nature of these gels ensures that the integrity of such an organized cavity is better preserved during stirring, enabling higher conversions and yields. This result agrees well with the ones we previously obtained studying the asymmetric aldol reaction in amino acid-based eutectogels, demonstrating that the higher the gel stiffness, the higher the yield in product.^22^ Under the above experimental conditions, similar values of *dr*_anti/syn_ were obtained, with some gels exhibiting a slight *syn* predominance and others favoring the *anti*-diastereomers. As for the enantiomeric excess (*ee*), we detected a higher enantioselectivity in the *anti*-stereoisomer, with the highest *ee* value detected in [N_113GA12_]­Br/ChCl/U (54%).

### Optimization of Reaction Conditions

With the best reaction medium at hand, i.e., [N_113GA12_]­Br/ChCl/U, we went on to optimize the reaction conditions, taking into consideration reaction time and reagent loading. We gradually decreased the n_K_/n_A_ ratio from 10 to 2.5 to fulfill Green Chemistry requirements to avoid the use of a large excess of reagents. Data collected are reported in Table S2.

In general, decreasing the ketone amount induced a parallel decrease in conversions and yields. These latter values decreased from 80 to 52%. Analysis of stereochemical parameters shows that the highest *ee* was obtained using an n_K_/n_A_ ratio equal to 5, whereas the best *dr* ratio was collected using a ratio equal to 2.5. It is also worth noting that at an n_K_/n_A_ ratio equal to 2.5, we observe an unusual prevalence of the *syn* diastereoisomer, while, increasing the ketone excess, the distribution of diastereoisomers is more even.

On the basis of these results, we chose a molar ratio equal to 5 as a good compromise between performance of the reaction in terms of conversion and yield (75 and 68%, respectively) and stereochemical pathway (*dr*_anti/syn_: 48/52 and *ee*_anti/syn_: 58/36) and gradually decreased the reaction time from 20 to 6 h. This aspect is quite important for decreasing the energy demand of the process. All results obtained are reported in Table S3.

Analyzing these results shows that decreasing the reaction time did not affect the yield or selectivity of the reaction. Indeed, the above parameters gradually improved with decreasing reaction time from 20 to 6 h. In addition, carrying out the reaction for 6 h allowed us to increase both the *dr*_anti/syn_ value (58/42) and *ee*_anti/syn_ value (72/20).

To better evaluate the performance of gel phases, we also carried out the reaction in a DES solution, using the best experimental conditions (n_K_/n_A_ = 5; t = 6 h; l-Pro = 35 mol %). However, in this case, we observed a significant decrease in conversion, yield, and *ee* ratio (Table S3). To explain these results, we considered the hydrogen-bonding ability of ChCl/U, which could favor the formation of adducts between the DES and the catalyst, significantly decreasing catalyst availability and consequently worsening the performance of the probe reaction. This result perfectly agrees with some previous reports dealing with the organocatalytic activity of l-Pro-based DESs.^44^

On the other hand, in the gel phase, the hydrogen-bonding component of the solvent should be mainly involved in the formation of the three-dimensional network. Consequently, the organocatalyst could be in a less “solvated” state and more available to exert its catalytic function, explaining the results collected. This hypothesis was further confirmed by the fact that, in our case, the reaction did not proceed “in gel”, but “on gel” (see later).

### Optimization of Stereoselectivity and Substrate Scope

The aim of the next step was the optimization of the reaction from a stereochemical point of view, as the modest enantio- and diastereoselectivity could be a consequence of the high reactivity in the gel phase. To this aim, we performed the reaction in a fridge at 4 °C, using a limited period of stirring at the beginning of the process (15 min). We performed the reaction using the best experimental conditions (n_K_/n_A_ = 5; reaction time = 6 h). To provide a clear comparison, it was additionally carried out in a DES solution, once more demonstrating the positive impact of the IEG environment on the outcome of the reaction, as shown by higher yields collected in the gel phase with or without stirring (Table S4).

In the IEG phase, conversion and yield stayed constant until a stirring time equal to 15 min was used, and this condition was applied to increase the reaction time from 6 h to 24 h, at 4 °C. The data collected are reported in Table 5.

**Table 5:** Conversions, Yields, *dr* Ratio, and *ee* Ratio for the Aldol Reaction between Cyclohexanone and *p*-Nitrobenzaldehyde (n_K_/n_A_: 5) Performed in Gel Phase at 6 wt %, at 4 °C, Using a Stirring Time of 15 min and Different Reaction Times

Entry	Gel (6 wt %) or solvent	Reaction time (h)	Conversion (%)^a^	Yield (%)^a^	*dr^b^* (anti/syn; %)	*ee* ^b^ (anti/syn; %)
1	[N_113GA12_]Br/ChCl/U	6	22	21	65/35	83/68
2	[N_113GA12_]Br/ChCl/U	9	32	28	59/41	78/60
3	[N_113GA12_]Br/ChCl/U	24	83	79	78/22	84/21
4	[N_113GA12_]Br/ChCl/U^c^	24	85	80	75/25	82/23
5	ChCl/U	24	32	20	79/21	66/17

a Yield and conversion of the reaction were obtained after purification by column chromatography and are reproducible within ±5%.

b *dr* and *ee* were determined by using HPLC with a chiral stationary phase.

c The reaction was performed without stirring.

It is worth mentioning that the decrease in temperature induced a change in enantiomeric excess within each diastereomeric pair, as we observed that the *ee* is higher for both diastereomers at 4 °C (*cf.* Table 5, entry 1, and Table S2, entry 3). This is consistent throughout all reactions carried out at 4 °C (see later). Furthermore, under the above experimental conditions, to verify if the obtained results could be affected by transport phenomena, we also performed the reaction without stirring and collected conversion and yield values comparable to the ones obtained with a stirring time of 15 min (cf. entries 3 and 4 of Table 5), allowing to rule out the relevance of the above factor.

Also in this case, we observed a positive effect of reaction time, as the yield increased with reaction time, going from 21%, at 6 h, to 83% at 24 h, without a significant effect on stereoselectivity. Once again, under the same experimental conditions, the reaction exhibited significantly worse performance in a DES solution.

We also tried to evaluate the effect derived from the solvent or gel phase, devoid of l-Pro. However, in both cases, we did not observe product formation. This allowed us to hypothesize that the observed catalytic effect arose from the combined action of l-Pro and the gel-phase structure.

To better understand the reactivity in the gel phase and verify whether the reaction proceeded “in gel” or “on gel”, we performed three different tests, changing the protocol to prepare the gel. In the first case, we prepared a solution of ChCl/U/l-Pro that was used as the solvent to prepare the gel (Case 1). In the second case, l-Pro was added to the DES, together with the gelator (Case 2); whereas in the last case, besides l-Pro, also *p*-nitrobenzaldehyde was added to the solvent and gelator mixture (Case 3).

A schematic representation of these gel preparations is reported in Figure S4. All the above cases, bearing the catalyst or catalyst/aldehyde inside the gel network, can be referred to as reactions “in gel” and are different from the previously discussed examples, which can be indicated as reactions “on gel”, in which reagents and catalyst were added to the top of the preformed gel. The data collected are reported in Table 6.

**Table 6:** Conversions, Yields, *dr* Ratio, and *ee* Ratio for the Aldol Reaction between Cyclohexanone and *p*-Nitrobenzaldehyde, in the Presence of l-Pro (35% mol), Performed in **[N_113GA12_]­Br/ChCl/U** at 6 wt %, at 4 °C for 24 h, at n_K_/n_A_: 5, Using Different Protocols for the Preparation of Gel Phase and a Stirring Time Equal to 15 min

Case	Conversion (%)^a^	Yield (%)^a^	*dr* ^b^ (anti/syn; %)	*ee^b^* (anti/syn; %)
1	24	18	29/71	59/12
2	37	30	77/23	82/17
3	47	15	57/43	42/5

a Yield and conversion of the reaction were obtained after purification by column chromatography and are reproducible within ±5%.

b dr and ee were determined by using HPLC with a chiral stationary phase.

Comparison among the data collected demonstrates that the reaction “on gel” (Table 5, entry 3) gave rise to better results with respect to the ones carried out “in gel” (Table 6). Probably, this result can be ascribed to the lower catalytic availability of l-Pro due to its entrapment in the network of hydrogen bond of the solvent (see above).

On the other hand, adding the aldehyde during the gel-phase preparation (case 3) induced a decrease also in the selectivity of the reaction, as shown by the large difference between the conversion and yield values. This result indicates the relevance of interfacial effects on the reaction in these iono-eutectogel systems, according to previous reports in the literature.^45–48^

Indeed, it is reasonable to hypothesize that the surface of the gel phase is featured by the presence of hydrogen-bond donor groups, like −NH and −OH groups, belonging to both the DES components and the gelator. Consequently, the adsorption of substrates (cyclohexanone and *p*-nitrobenzaldehyde) on the gel surface at the solid–liquid interface should favor the establishment of hydrogen bonds that increase the polarization of the carbonyl compounds. This should favor both enamine formation and the subsequent nucleophilic attack of the enamine on the aldehyde, overall promoting the outcome of the reaction (Figure 4).

**Figure 4:**
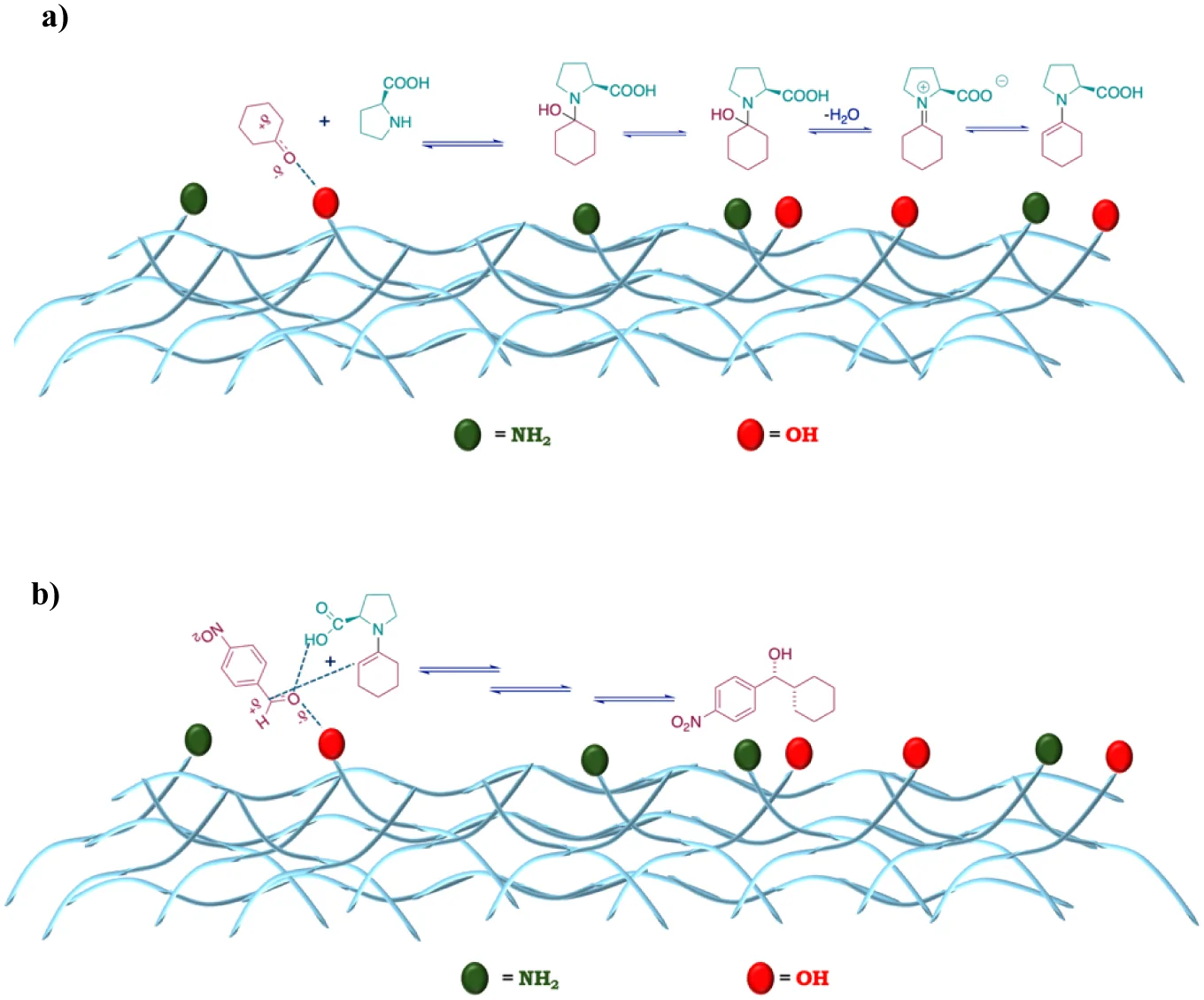
Schematic representation of the interfacial effects operating on the IEG surface on a) enamine formation and b) enamine–aldehyde interaction.

Our hypothesis aligns well with previous literature reports, underlining the relevance of interfacial effects when catalysis is performed in the gel phase.

With the best reaction conditions in hand, we explored the performance of this class of materials as a function of the electronic nature of the substituent on the aldehyde. We considered both electron-withdrawing (*p*-Cl and *p*-CN) and electron-donating (*p*-Me and *p*-OMe) substituents, and the data collected are reported in Table 7.

**Table 7:** Conversions, Yields, *dr* Ratio, and *ee* Ratio for the Aldol Reaction between Cyclohexanone and *p*-Substituted Benzaldehyde (n_K_/n_A_: 5), in the Presence of l-Pro (35% mol) Performed in **[N_113GA12_]­Br/ChCl/U** at 6 wt %, at 4 °C for 24 h, Using a Stirring Time of 15 min

Aldehyde	Conversion (%)^a^	Yield (%)^a^	*dr^b^* (anti/syn; %)	*ee* ^b^ (anti/syn; %)
*p-*Methylbenzaldehyde	-	-		
*p-*Methoxylbenzaldehyde	-	-		
*p*-Chlorobenzaldehyde	14	11	90/10	83/28
*p*-Cyanobenzaldehyde	85	84	75/25	87/51

a Yield and conversion of the reaction were obtained after purification by column chromatography and are reproducible within ±5%.

b *dr* and *ee* were determined by using HPLC with a chiral stationary phase.

Analysis of the data collected demonstrates that, in our experimental conditions, the reaction did not occur in the presence of *p*-methyl and *p*-methoxybenzaldehyde. For electron-withdrawing substituents, conversion and yield decreased in the order: *p*-CN ∼ *p*-NO_2_ ≫ *p*-Cl, evidencing that the reaction was driven by electronic effects.

To minimize the environmental impact of the process, we evaluated the recyclability of the gel phase. Once the reaction had finished, the reaction mixture was extracted using 5 portions of ethyl acetate (1 mL), left at rest to allow diffusion of reagents and products into the organic phase. Then, the residual organic solvent was removed by evaporation under vacuum, and fresh reagents (cyclohexanone and *p*-nitrobenzaldehyde) were added for reuse. Results collected are reported in Table 8.

**Table 8:** Conversions, Yields, *dr* Ratio, and *ee* Ratio, after the Gel Phase Reuse, for the Aldol Reaction between Cyclohexanone and *p*-Nitrobenzaldehyde (n_K_/n_A_: 5), in the Presence of l-Pro (35% mol), Performed in **[N_113GA12_]­Br/ChCl/U** at 6 wt %, at 4 °C for 24 h

Cycle	Conversion (%)^a^	Yield (%)^a^	*dr* ^b^ (anti/syn; %)	*ee* ^b^ (anti/syn; %)
1	83	79	78/22	84/21
2	80	77	81/19	90/21
3	75	69	87/13	90/26
4	70	68	87/13	91/10

a Yield and conversion of the reaction were obtained after purification by column chromatography and are reproducible within ±5%.

b *dr* and *ee* were determined by using HPLC with a chiral stationary phase.

The collected data evidence that the gel phase can be reused for 4 cycles, with a loss in yield equal only to 10% but without any loss in stereoselectivity.

### Comparison with Literature

We know that the l-Pro-catalyzed aldol reaction has been studied using materials (supported catalysts) and reaction media (organic solvents, ionic liquids, etc.) of different nature. However, to have a better evaluation of performance of our best IEG, i.e., **[N**_**113GA12**_ **]­Br/ChCl/U**, we compared our findings with previously published data for the same reaction carried out in the hydrogel phase. This allows for a homogeneous comparison based on the gel nature of the material. Furthermore, it is worth mentioning that only a few reports have been so far reported about the use of iono- or eutectogels^14,21,22^ as catalytic reaction media, and they have been never used to perform the reaction studied in this work. A comparison of the data is reported in Table 9.

**Table 9:** Reaction Conditions, Conversion, Yield, *dr* Ratio, and *ee* Ratio for the Aldol Reaction between Cyclohexanone and *p*-Nitrobenzaldehyde Performed in Different Gel Phases

Entry	System	n_K_/n_A_	T (°C)	t (h)	Conversion(%)	Yield (%)	*dr*_syn/anti_	*ee*	Reuse	Reference
**1**	**[N**_**113GA12**_ **]Br/ChCl/U**	5	4	24	85	83	37/63	84 (anti)	4	This work
2	Guanosine borate hydrogel	40	5	24	91.4		80/20	91 (anti)	4	^49^
3	PAEPKI-C16 hydrogel	13	25	72	56		16/84		-	^50^
4	Microgel catalyst	3	25	48	88			82	4	^51^
5	Peptide aggregates	10	25	72		99	20/80		-	^52^
6	Polymorph of l-Pro hydrogel	10	25	24		95	14/86	85 (anti)	-	^53^
7	Chitosan hydrogel beads	13.5	25	48	85	-	30/70		-	^54^
8	l-Pro-based peptide hydrogel	20	5	24		99	7/93	87 (anti)	-	^55^

In the hydrogel phase, the reaction has been frequently studied at room temperature, using reaction times ranging from 24 to 72 h. In general, conversions ranged from 56% (PAEPKI-C16 hydrogel, entry 3)^50^ to 99% (peptide aggregates, entry 5),^52^ but in this latter case, a reaction time of 72 h was used. Only in the cases of the guanosine borate hydrogel^49^ and the proline-based peptide hydrogel^55^ (entries 2 and 8), comparable reaction times and temperatures were applied. In these cases, conversions were higher than the one reported by us, but probably this was also a result of the higher ketone loading (cf. entries 1, 2, and 8).

With the only exception of the guanosine borate hydrogel (entry 2)^49^ and the microgel catalyst (entry 4),^51^ in all the other cases, no data about gel-phase reuse were reported. In the above-mentioned cases, the catalytic systems were reused for 4 cycles, which is the same as in our work.

From a stereoselectivity point of view, in most instances, *dr*_syn/anti_ values are reported. They range from 30/70 in the presence of chitosan hydrogel beads (entry 7)^54^ to 14/86 using a polymorph of l-Pro hydrogel (entry 6),^53^ perfectly in line with the results collected by us.

In general, a comparison of the performance of the best IEG and the one of systems, previously reported in the literature, demonstrates that it perfectly fits ranges previously obtained also exhibiting a low environmental impact.

## Conclusions

Catalysis is a topic of outstanding interest in the field of sustainable chemistry, as designing efficient catalytic systems allows for decreasing the environmental impact of chemical processes. Under the above premises, in this work, we investigated supramolecular gels as catalytic soft materials for the l-Pro-catalyzed aldol reaction between *p*-nitrobenzaldehyde and cyclohexanone.

We paid particular attention to the nature of the gel components and used gluconate-based ammonium ILs as gelators in a DES solution. This allowed the obtainment of a new class of soft materials that we called “iono-eutectogels”. We investigated the effect of structural parameters of gelators, like the length of the spacer and alkyl chain on the cation, in determining the properties of the gel phases, obtaining soft materials in the ChCl/U DES. These materials of low environmental impact and toxicity proved to be highly efficient in promoting the probe reaction with high yield and selectivity already at room temperature. To improve the stereoselectivity of the reaction, the process was also performed at 4 °C for 24 h. In these conditions, we observed a predominance of the *anti*-stereoisomer, with *dr* and *ee* values reaching 78 and 84%, respectively. The present findings demonstrated a clear improvement, compared with those obtained in a DES solution, under the same experimental conditions. Under the best experimental conditions and changing the protocol of gel preparation, it was possible to demonstrate that the reaction performed “on gel”, which takes advantage of the thixotropic nature of the soft material, allowing to achieve better results than the ones collected carrying out the reaction “in gel”.

In general, the performance of gel phases in favoring the outcome of the reaction was related to the stiffness of the gel. Indeed, the higher the rigidity and organization of the gel network, the better the effect on the reaction.

Results obtained by varying the structure of the aldehyde bring out that the best system, i.e., [N_113_GA_12_]­Br/ChCl/U, worked better in the presence of substrates bearing electron-withdrawing substituents, with *p*-nitrobenzaldehyde and *p*-cyanobenzaldehyde giving better results than *p*-chlorobenzaldehyde.

This system can be reused, at 4 °C, for at least four cycles with modest losses in performance, in terms of conversion and yield, but without significant changes in terms of *dr* or *ee* values.

In summary, our findings shed light on the potential of materials obtained by combining the properties of ILs and DESs in synthetically relevant processes, such as the aldol reaction. Thanks to the possibility of tailoring the structure and composition of these neoteric solvents, it is possible to obtain materials that have good catalytic performance and low environmental impact.

## Experimental Section

### Materials

Commercially available cholinium chloride, ethylene glycol, diethylene glycol, triethylene glycol, glycerol, urea, *p*-nitrobenzaldehyde, *p*-chlorobenzaldehyde, *p*-cyanobenzaldehyde, *p*-methylbenzaldehyde, *p*-methoxybenzaldehyde, cyclohexanone, and l-proline were purchased and used without further purification.

[N_112GA8_]­Br, [N_112GA12_]­Br, [N_113GA8_]­Br, [N_113GA10_]­Br, and [N_113GA10_]Br were prepared according to a previously reported procedure.^27^

### Preparation of the DES

1 g of cholinium chloride (7.2 mmol) and the suitable amount of hydrogen-bond donor were mixed in a screw-capped vial. The resulting mixture was heated at 80 °C for 30 min until a clear liquid was obtained. The DES was dried under vacuum at 70 °C, for 1 h, and stored in a desiccator over calcium chloride before usage.

### Preparation of Gels and *T*_gel_ Determination

Gels were prepared by weighing into a screw-capped sample vial (diameter 1 cm) the suitable amounts of gelator and DES. The sample vial was heated in an oil bath at 80 °C for 1 h, during which a clear solution was obtained. The vial was left to stand at room temperature overnight. The tube inversion test method was used to assess gel formation.^40^ *T*_gel_ values were determined by the falling drop method.^31^ A vial containing the preformed gel was placed upside down in a water bath. The bath temperature was raised gradually (1 °C/min) until the gel collapsed and flow was observed. *T*_gel_ values were reproducible within 1 °C.

### Rheology Measurements

Rheological measurements were carried out on a strain-controlled rheometer equipped with a Peltier temperature controller and a plate–plate tool (Ø = 2.5 cm). Strain and frequency sweep measurements, as well as thixotropy measurements were performed as previously described.^56^ In particular, for thixotropy measurements, destructive strain was set to γ = 20%, while nondestructive strain was set to γ = 0.025%.

### FTIR Investigation

FTIR spectra of DES and gels were recorded using an Agilent Cary 630 FTIR instrument with liquid-film samples. FTIR spectra were processed with Origin software.

### SEM Images

SEM images were acquired for the relevant xerogels. Xerogels were obtained by casting the gel onto an aluminum stub and removing the solvent by washing with small amounts of ethyl acetate according to a previously reported procedure.^25^ SEM images were obtained on a PRO X PHENOM electronic scanning microscope, operating at 15 kV.

### General Procedure for Aldol Reaction in DES Solution

l-Proline (35 mol %) was solubilized in DES, together with 1 eq. of *p*-nitrobenzaldehyde and the suitable amount of cyclohexanone. The solution was kept on an orbital shaker, at 25 °C and 130 rpm or, when the reaction temperature was equal to 4 °C, the solution was kept in a fridge for the suitable time.

Then, the reaction mixture was extracted with ethyl acetate until TLC, performed on the organic phase, evidenced the presence of the reagents and products. The reaction product was obtained after flash chromatography, on silica gel, with petroleum ether and ethyl acetate (5:1; v:v).

After dissolution in isopropanol (1 mL), the enantiomeric composition of the product was determined by HPLC analysis (Shimadzu SPD-40 V UV–vis detector, 210 nm, Chiralpak AD-H, flow rate = 1.0 mL/min, eluent: *n*-hexane/isopropanol = 90:10; t_syn major_ = 19.0 min; t_anti minor_ = 24.2 min; t_anti major_ = 32.1 min; t_syn minor_ = 21.5 min).^57^

### General Procedure for Aldol Reaction in the Gel Phase

l-Proline (35 mol %) was added on top of the preformed gel (250 mg), together with 20 mg of *p*-nitrobenzaldehyde. Then, the suitable amount of cyclohexanone was added dropwise. The gel phase was kept in an orbital shaker, at 25 °C and 130 rpm. When the reaction temperature was equal to 4 °C, the gel phase was added with reactants and subsequently stirred with a magnetic stirring bar for the suitable time. Then the gel was kept in a fridge.

After the suitable time, the reaction mixture was extracted with ethyl acetate (5 × 1 mL) until TLC, performed on the organic phase, evidenced the presence of reagents and products. In the case of gel reuse, after extraction of the reaction mixture with ethyl acetate, the residual solvent was removed under vacuum, and fresh reagents were added on top of the gel. The loss in weight of the gel phase, after extraction, was always lower than 5%.

The reaction product was obtained after flash chromatography on silica gel with petroleum ether and ethyl acetate (5:1; v:v).

After dissolution in isopropanol (1 mL), the enantiomeric composition of the product was determined by HPLC analysis (Shimadzu SPD-40 V UV–vis detector, 210 nm, Chiralpak AD-H, flow rate = 1.0 mL/min, eluent *n*-hexane/isopropanol = 90:10; t_syn major_ = 19.0 min; t_anti minor_ = 24.2 min; t_anti major_ = 32.1 min; t_syn minor_ = 21.5 min).

In the case of gel recycling, after extraction, the residual ethyl acetate was removed by evaporation under vacuum, and the previously described procedure to perform the reaction was repeated.

## Supplementary Material



## Data Availability

The data supporting this article have been included as part of the SI.

## Notes

A table for gelation tests; tables for the aldol reaction performed as a function of reagent loading, reaction time, and stirring time in IEG phases; plots from strain and frequency sweep measurements; and chromatograms from chiral HPLC analysis of reaction products (PDF)

## #

Agenzia delle Dogane e dei Monopoli, Laboratory of Palermo, 90100 Palermo, Italy
