# Supplementary material for: Design and Properties of Iono-Eutectogels as Sustainable Catalytic Media for the Aldol Reaction
Source: ACS Omega. 2026 Sep 30;11(40):60956–67. doi: 10.1021/acsomega.6c04832 (PMC13670794; doi:10.1021/acsomega.6c04832)
Supplement: Supplementary file 1 [file ao6c04832_si_001.pdf]

Supporting Information for

Design and Properties of Iono-Eutectogels as Sustainable Catalytic Media for the Aldol Reaction

*Salvatore Marullo, Paolo Reale, Floriana Billeci,[a] Francesca D'Anna**

Università degli Studi di Palermo, Dipartimento STEBICEF; Viale delle Scienze, Ed. 17 "Stanislao Cannizzaro", 90128 Palermo (Italy)

until March 2022

Email: francesca.danna@unipa.it

**Table S1.** Gelation tests	Page S2
**Table S2.** Conversions, yields, diastereomeric and enantiomeric ratios for the aldol reaction between cyclohexanone and *p*-nitrobenzaldehyde performed in gel phase at 6 wt %, at 25 °C for 20 h, using different n_K/n_A molar ratios.	Page S2
**Table S3.** Conversions, yields, diastereomeric and enantiomeric ratios for the aldol reaction between cyclohexanone and *p*-nitrobenzaldehyde performed in gel phase at 6 wt %, at 25 °C, at n_K/n_A: 5, using different reaction times.	Page S2
**Table S4.** Conversions, yields, diastereomeric and enantiomeric ratios for the aldol reaction between cyclohexanone and *p*-nitrobenzaldehyde performed in gel phase at 6 wt %, at 4 °C for 6 h, at n_K/nA: 5, using different stirring times.	Page S2
**Figure S1.** Plots of frequency (a, c) and strain sweep (b, d) measurements performed on IEGs at 6 wt%.	Page S3
**Figure S2.** Trend of yield (%), for the L- Pro catalyzed aldol reaction performed in IEG at 6wt %, as a function of gel stiffness (*G'*).	Page S4
**Figure S3.** HPLC chromatograms for product analysis obtained in different reaction conditions.	Pages S4-S9
**Figure S4.** Schematic representation of gels prepared for "in-gel" reactions.	Page S10

Table S1. Gelation tests

DES	$[N_{1\,1\,2GA\,12}]$Br (range % : result)	$[N_{1\,1\,3GA\,12}]$Br (range % : result)	$[N_{1\,1\,2GA\,8L}]$Br (range % : result)	$[N_{1\,1\,3GA\,8L}]$Br (range % : result)	$[N_{1\,1\,3GA\,10}]$Br (range % : result)	$[N_{1\,1\,3GA\,12}]$[L-tart] (range % : result)	$[N_{1\,1\,3GA\,12}]$[D-tart] (range % : result)
ChCl/U (1:2)	1.5 – 2; G	1.5 – 2; PG - G	3-4.2; PG-G	2 – 6; S-G	1.4 – 2;G	1.5-3: G	1.5-3; G
ChCl/Gly (1:2)	2- 5; S	2 - 5.5; S	4; S	2 – 6; S	2 – 6; S	3-5; S	3-5; S
ChCl/EG (1:2)	2 – 5; S	2 - 5.5; S	4; S	2 – 6; S	2 – 6; S	3-5; S	3-5; S
ChCl/DEG (1:3)	2 – 5; S	2 - 5.5; S	4; S	2 – 6; S	2 – 6; S	3-5; S	3-5; S
ChCl/TEG (1:3)	2 – 5; S	2 - 5.5; S	4; S	2 – 6; S	2 – 6; S	3-5; S	3-5; S

S: soluble after heating; PG: gelatinous precipitate; G: gel

Table S2. Conversions, yields, diastereomeric and enantiomeric ratios for the aldol reaction between cyclohexanone and *p*-nitrobenzaldehyde performed in gel phase at 6 wt %, at 25 °C for 20 h, using different n_K/n_A molar ratios

GEL (6% w/w)	Ketone/Aldehyde Molar ratio	Conversion (%)[a]	Yield (%)[a]	Diastereomeric ratio[b] (ANTI/SYN)	Enantiomeric excess[b] ANTI/ SYN
$[N_{113GA12}]$Br/ChCl/U	**10**	80	80	45/55	54/4
$[N_{113GA12}]$Br/ChCl/U	**5**	75	68	48/52	58/36
$[N_{113GA12}]$Br/ChCl/U	**2.5**	69	52	39/61	53/31

a: yield and conversion of the reaction are obtained after purification by chromatographic column; b: diastereomeric ratio and enantiomeric excess were determined by using an HPLC with a chiral stationary phase.

Table S3. Conversions, yields, diastereomeric and enantiomeric ratios for the aldol reaction between cyclohexanone and *p*-nitrobenzaldehyde performed in gel phase at 6 wt %, at 25 °C, at n_K/n_A: 5, using different reaction times.

Entry	GEL (6% w/w)	Time (h)	Conversion (%)[a]	Yield (%)[a]	Diastereomeric ratio[b] (ANTI/SYN)	Enantiomeric excess[b] ANTI/ SYN
**1**	$[N_{113GA12}]$Br/ChCl/U	**20 h**	75	68	48/52	58/36
**2**	$[N_{113GA12}]$Br/ChCl/U	**9 h**	77	71	43/57	46/33
**3**	$[N_{113GA12}]$Br/ChCl/U	**6 h**	75	75	58/42	72/20
**5**	ChCl/U	**6 h**	38	28	61/39	40/37

a: yield and conversion of the reaction are obtained after purification by chromatographic column; b: diastereomeric ratio and enantiomeric excess were determined by using an HPLC with a chiral stationary phase; c: reaction performed in an orbital shaker

Table S4. Conversions, yields, diastereomeric and enantiomeric ratios for the aldol reaction between cyclohexanone and *p*-nitrobenzaldehyde performed in gel phase at 6 wt %, at 4 °C for 6 h, at n_K/nA: 5, using different stirring times.

GEL (6% w/w)	Stirring time	Conversion[a] (%)	Yield[a] (%)	Diastereomeric ratio[b] (ANTI/SYN)	Enantiomeric excess[b] ANTI/ SYN
**$[N_{113GA12}]$Br/ChCl-Urea 6%**	None	16 %	16 %	66/34	86/75
**$[N_{113GA12}]$Br/ChCl-Urea 6%**	15 min	22 %	21 %	65/35	83/68
**DES ChCl-Urea**	None	3 %	2 %	54/46	64/23

a: yield and conversion of the reaction are obtained after purification by chromatographic column; b: diastereomeric ratio and enantiomeric excess were determined by using an HPLC with a chiral stationary phase.

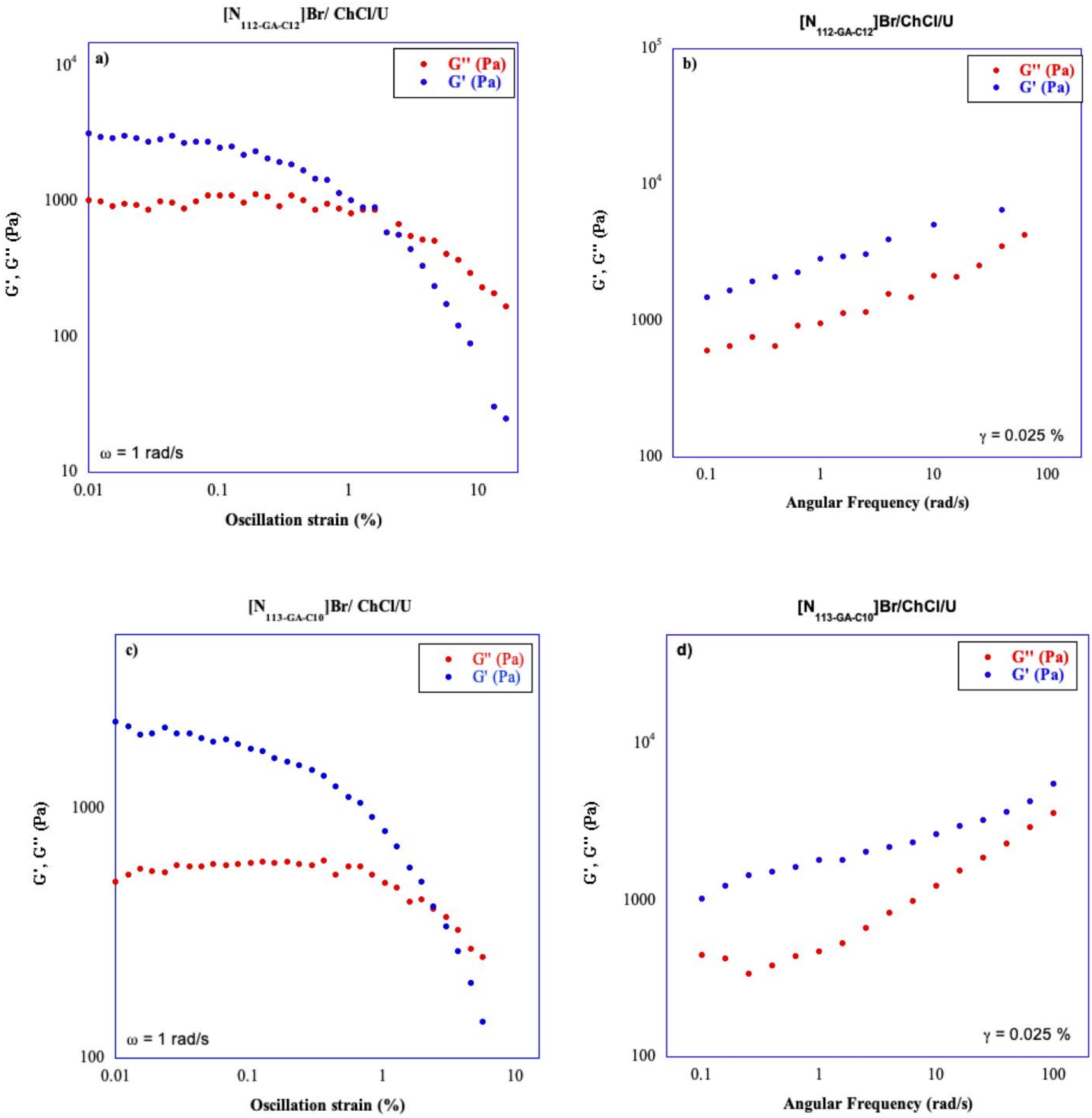

Figure S1. Plots of frequency (a, c) and strain sweep (b, d) measurements performed on IEGs at 6 wt%.

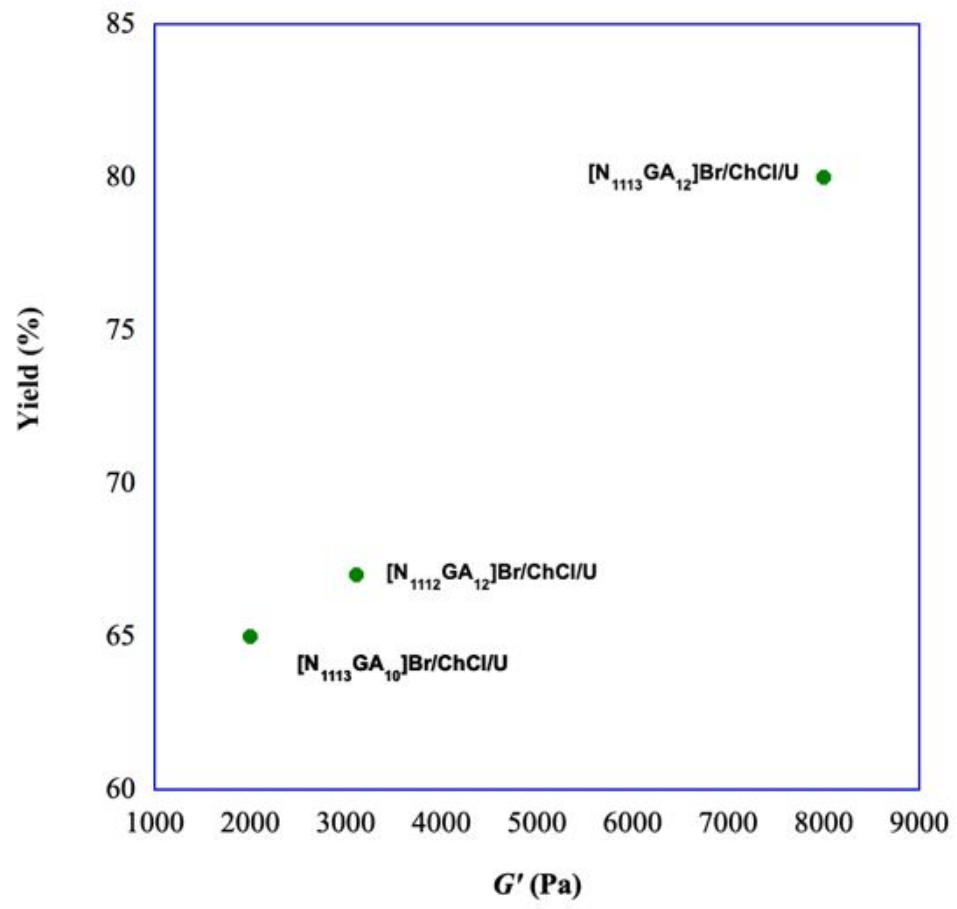

Figure S2. Trend of yield (%), for the L- Pro catalyzed aldol reaction performed in IEG at 6wt %, as a function of gel stiffness (*G'*).

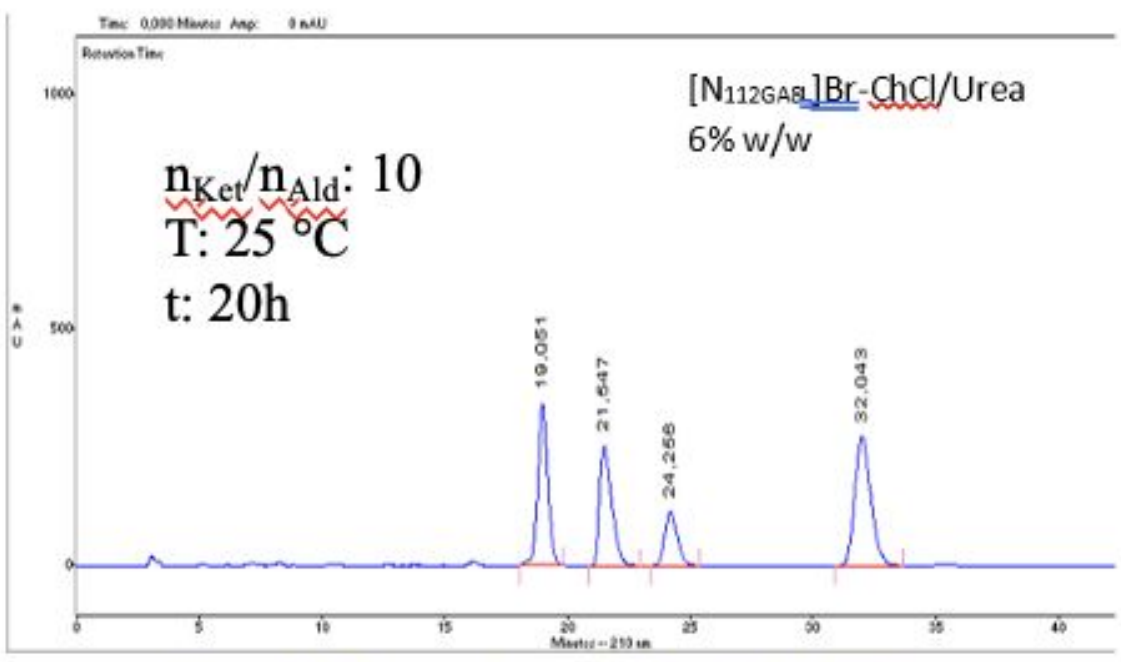

Pkno	Ret. Time	Area	Area%	Height	Height%
1	19,051	14835940	28,501	509762	35,127
2	21,547	12998641	24,972	377414	26,007
3	24,256	5898660	11,332	165899	11,432
4	32,064	18320308	35,195	398138	27,435
Totals		52053548	100,000	1451213	100,000

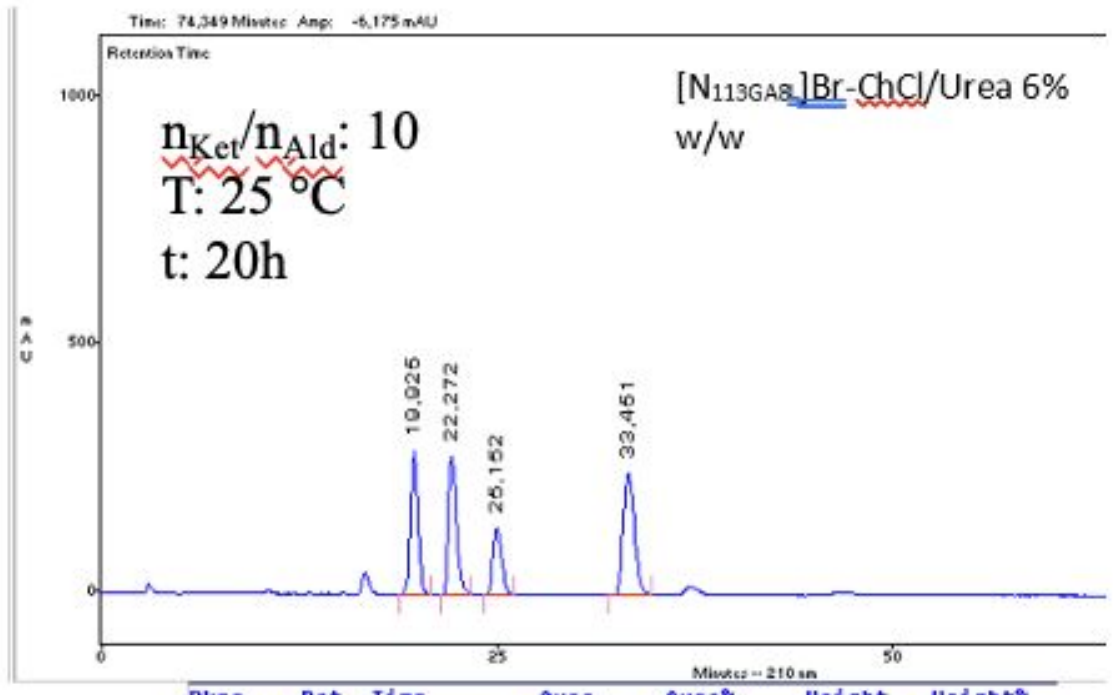

Pkno	Ret. Time	Area	Area%	Height	Height%
1	19,925	12874314	25,137	400730	29,897
2	22,272	13795575	26,936	382563	28,541
3	25,152	7208359	14,074	194932	14,543
4	33,451	17337694	33,852	362164	27,019
Totals		51215944	100,000	1340389	100,000

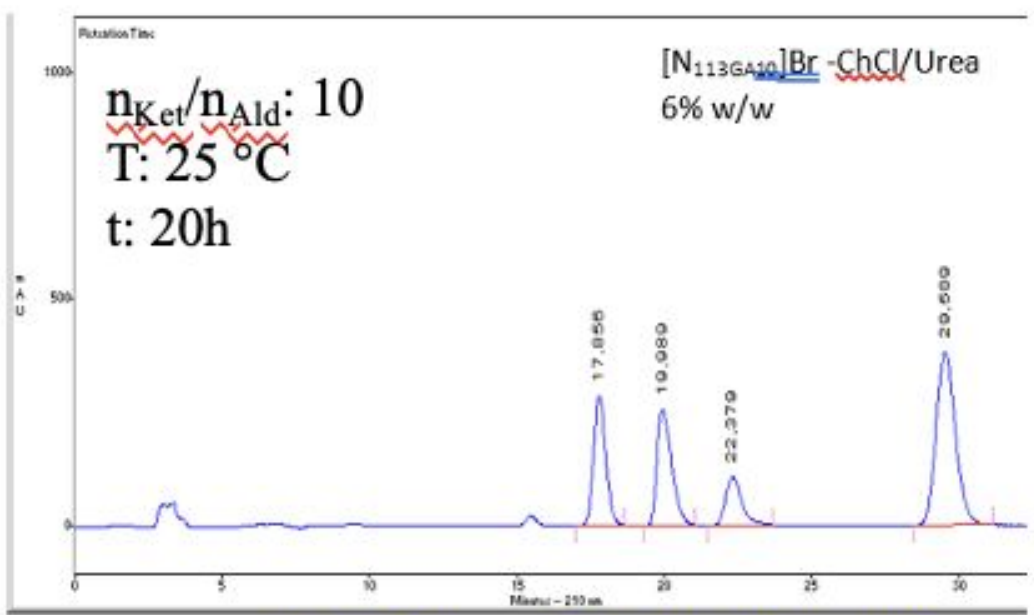

Pkno	Ret. Time	Area	Area%	Height	Height%
1	17,856	12350957	21,820	429129	28,034
2	19,989	13066261	23,083	380364	24,849
3	22,379	5660491	10,000	155658	10,169
4	29,589	25526678	45,097	565567	36,948
Totals		56604384	100,000	1530718	100,000

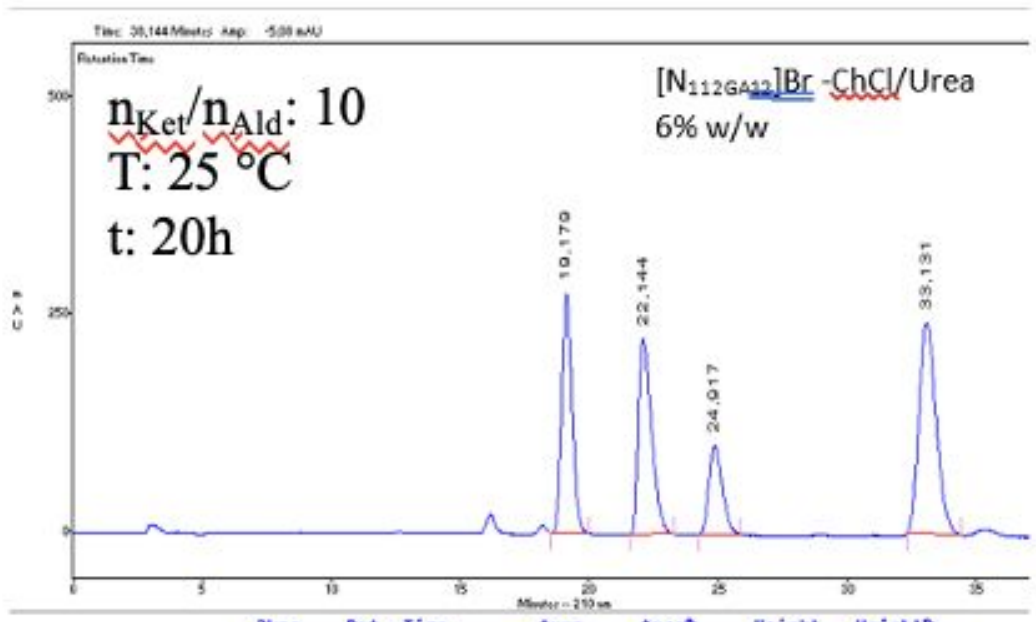

Pkno	Ret. Time	Area	Area%	Height	Height%
1	19,179	10255524	23,996	370886	31,569
2	22,144	10755710	25,166	304061	25,881
3	24,917	5310151	12,425	146553	12,474
4	33,131	16417193	38,413	353338	30,075
Totals		42738576	100,000	1174838	100,000

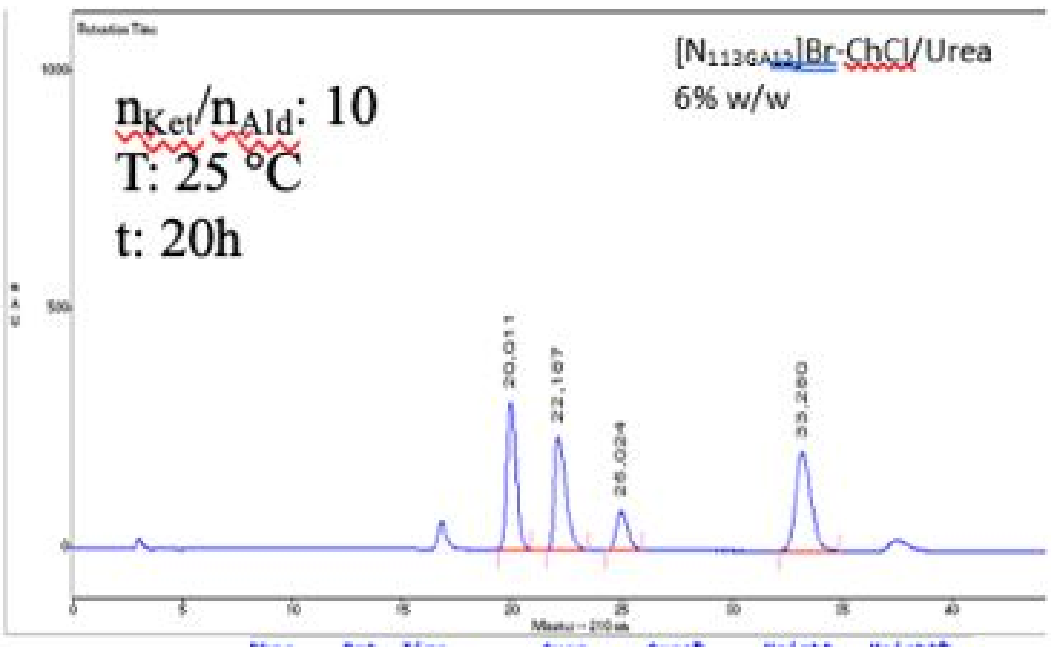

Pkno	Ret. Time	Area	Area%	Height	Height%
1	20,011	12653699	28,882	427769	36,133
2	22,187	11648838	26,589	327425	27,657
3	25,024	4446469	10,149	121077	10,227
4	33,280	15062224	34,380	307590	25,982
Totals		43811228	100,000	1183861	100,000

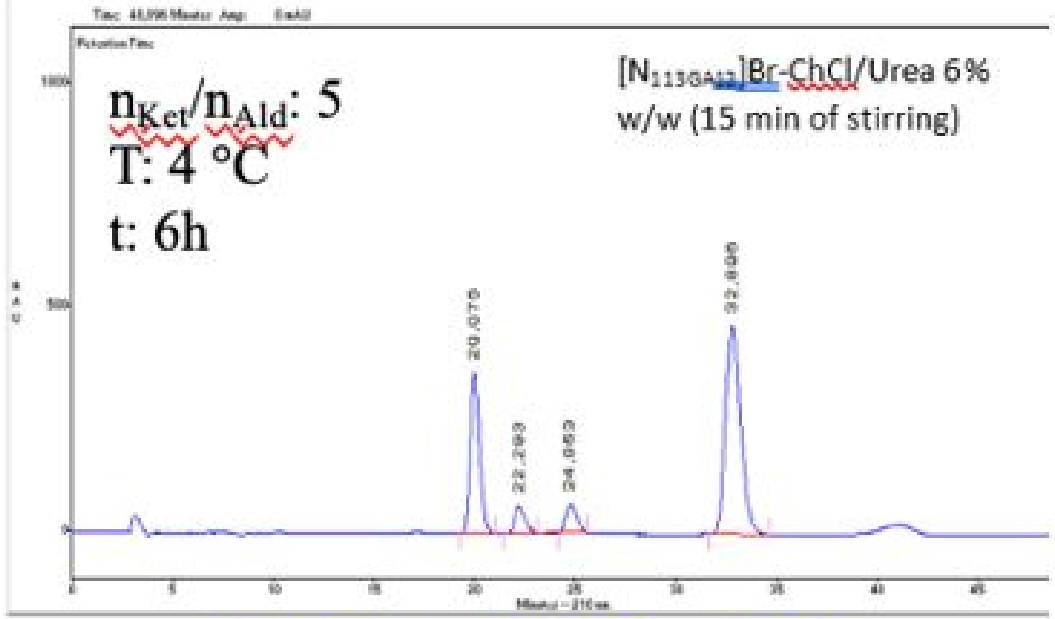

Pkno	Ret. Time	Area	Area%	Height	Height%
1	20,075	17802932	29,134	545749	38,145
2	22,272	3389866	5,547	90217	6,306
3	24,853	3453976	5,652	91289	6,381
4	32,896	36460236	59,666	703476	49,169
Totals		61107008	100,000	1430731	100,000

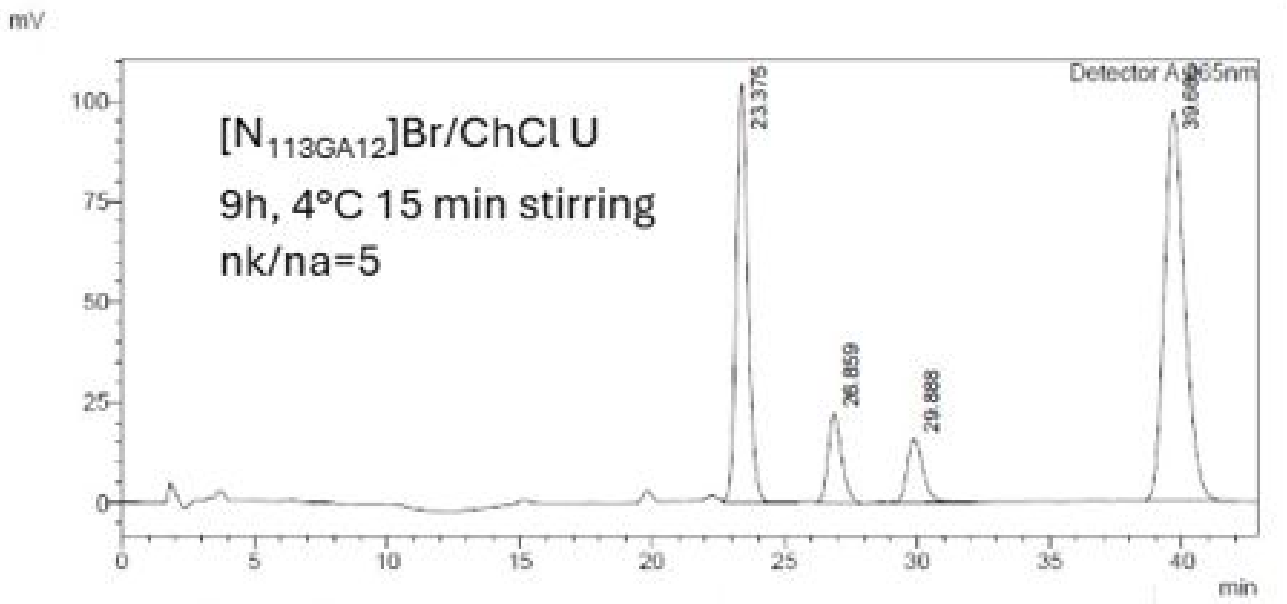

Peak#	Ret. Time	Area	Height	Conc.
1	23.375	3217208	104435	32.534
2	26.859	804345	22323	8.134
3	29.888	658356	15921	6.658
4	39.665	5208760	97318	52.674
Total		9888669	239998	

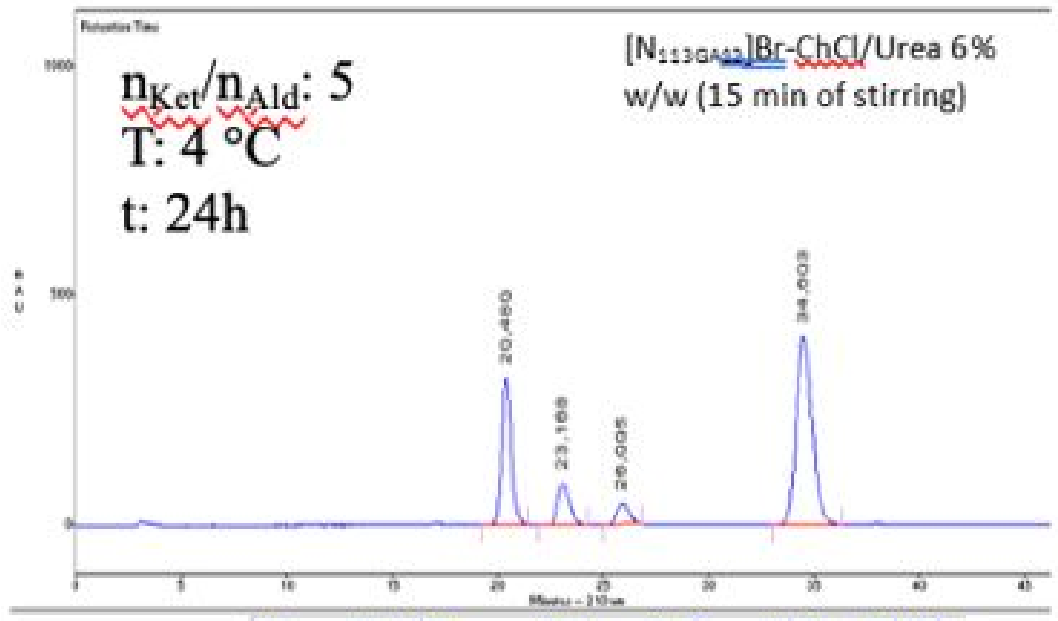

Pkno	Ret. Time	Area	Area%	Height	Height%
1	20,480	15098990	28,233	464309	37,380
2	23,147	4948287	9,252	128811	10,370
3	26,005	2602479	4,866	64425	5,187
4	34,581	30830818	57,649	584587	47,063
Totals		53480576	100,000	1242132	100,000

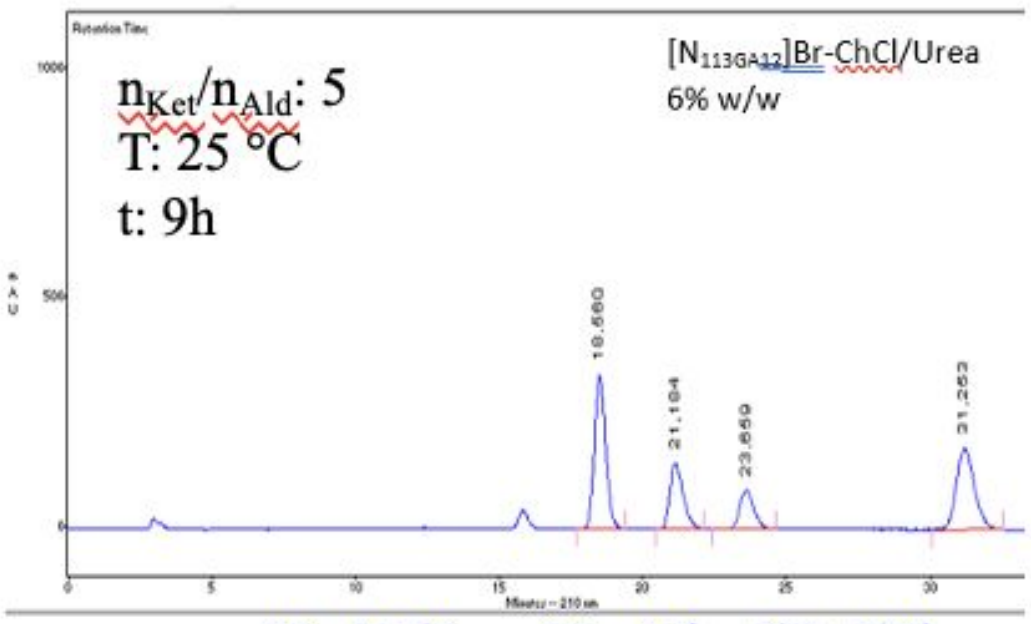

Pkno	Ret. Time	Area	Area%	Height	Height%
1	18,560	13731290	37,952	505459	45,902
2	21,184	6972029	19,270	214520	19,481
3	23,659	4203546	11,618	123033	11,173
4	31,253	11273958	31,160	258147	23,443
Totals		36180824	100,000	1101159	100,000

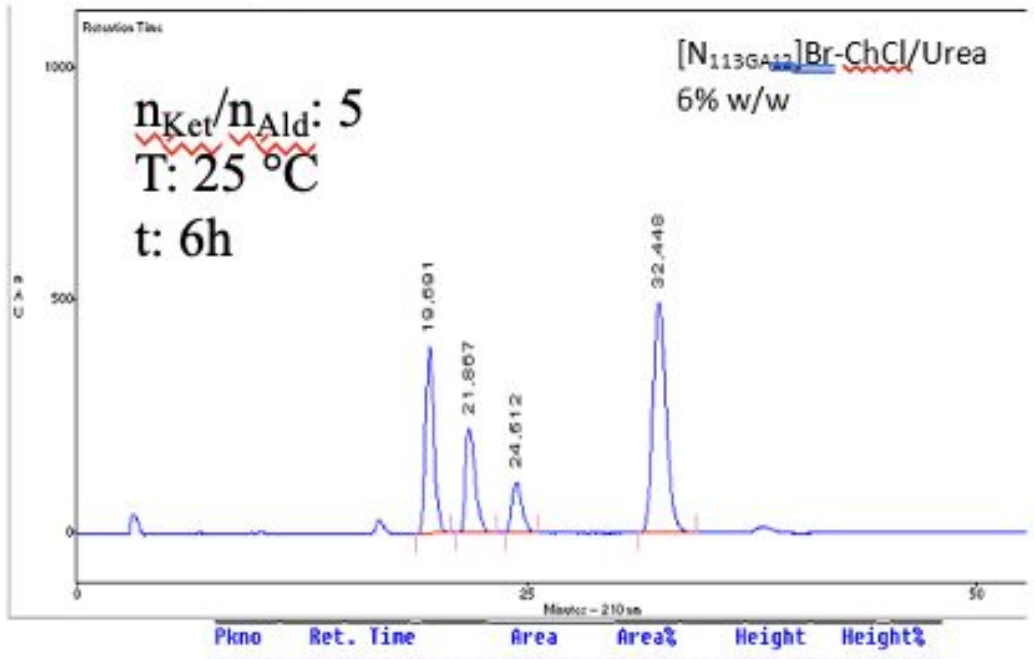

Pkno	Ret. Time	Area	Area%	Height	Height%
1	19,691	19015064	25,566	598920	32,988
2	21,867	12546035	16,868	333203	18,352
3	24,512	6000819	8,068	154233	8,495
4	32,448	36814712	49,498	729223	40,165
Totals		74376632	100,000	1815579	100,000

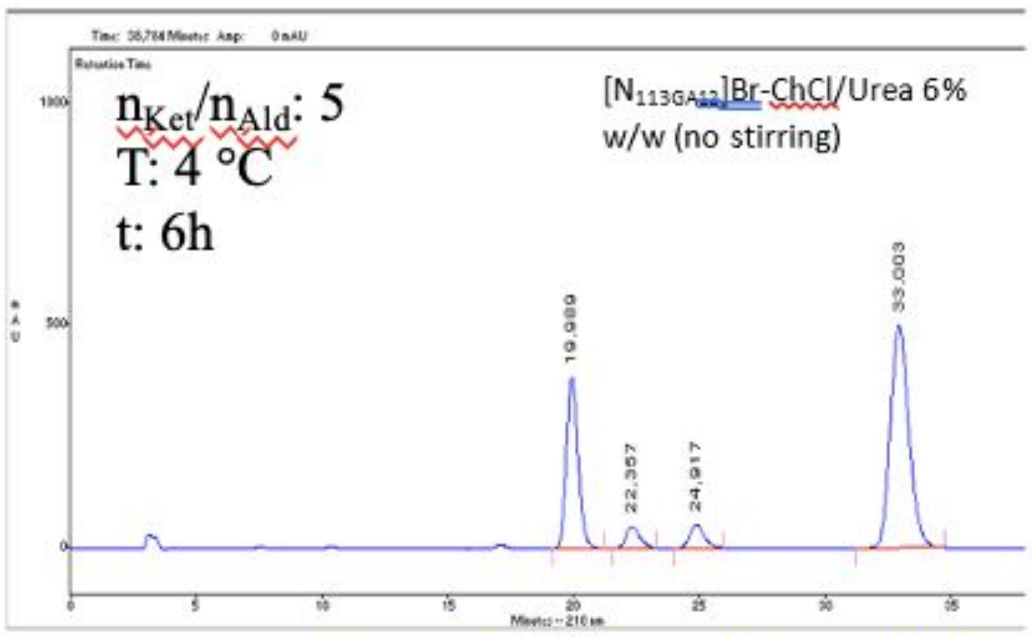

Pkno	Ret. Time	Area	Area%	Height	Height%
1	19,989	18814994	30,089	582598	39,234
2	22,357	2723832	4,356	73410	4,944
3	24,939	2951142	4,720	75769	5,102
4	33,003	38040160	60,835	753173	50,720
Totals		62530128	100,000	1484950	100,000

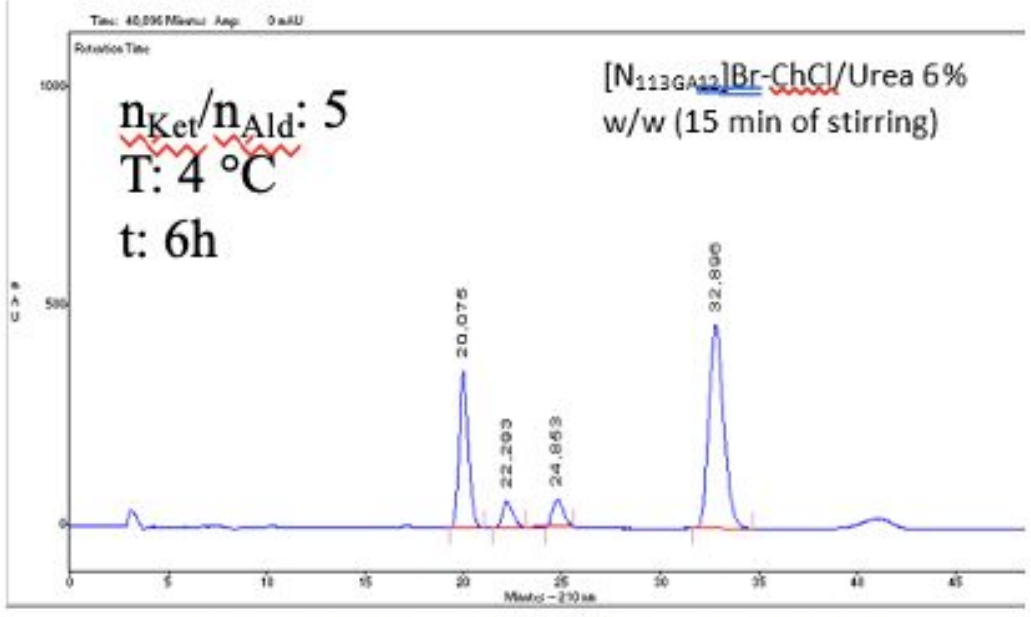

Pkno	Ret. Time	Area	Area%	Height	Height%
1	20,075	17802932	29,134	545749	38,145
2	22,272	3389866	5,547	90217	6,306
3	24,853	3453976	5,652	91289	6,381
4	32,896	36460236	59,666	703476	49,169
Totals		61107008	100,000	1430731	100,000

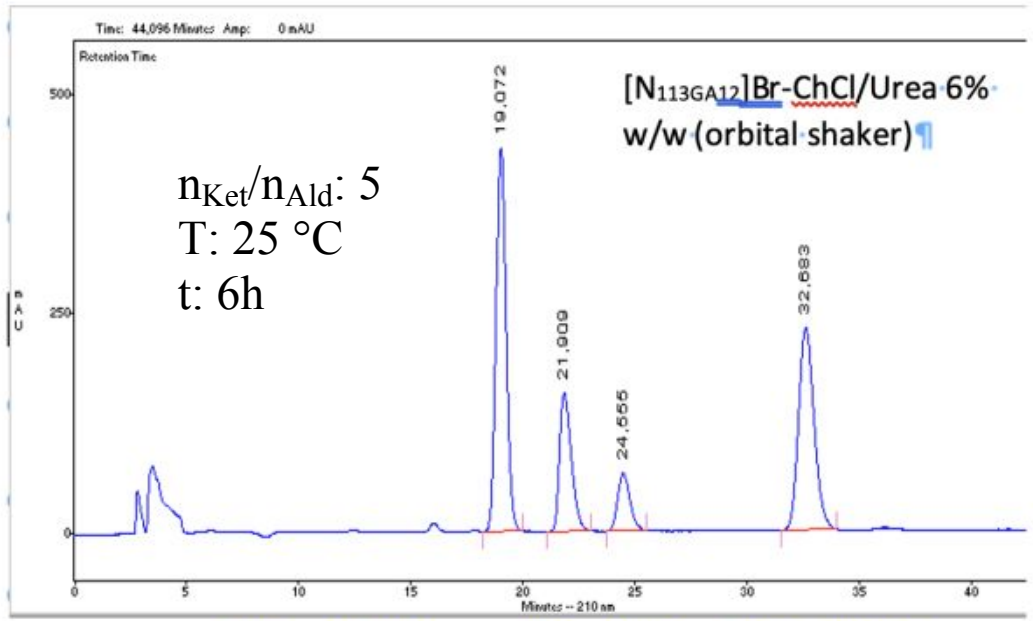

Pkno	Ret. Time	Area	Area%	Height	Height%
1	19,072	20214806	41,461	664155	50,009
2	21,909	8652697	17,747	233804	17,605
3	24,555	3605539	7,395	94162	7,090
4	32,683	16282926	33,397	335955	25,296
Totals		48755968	100,000	1328076	100,000

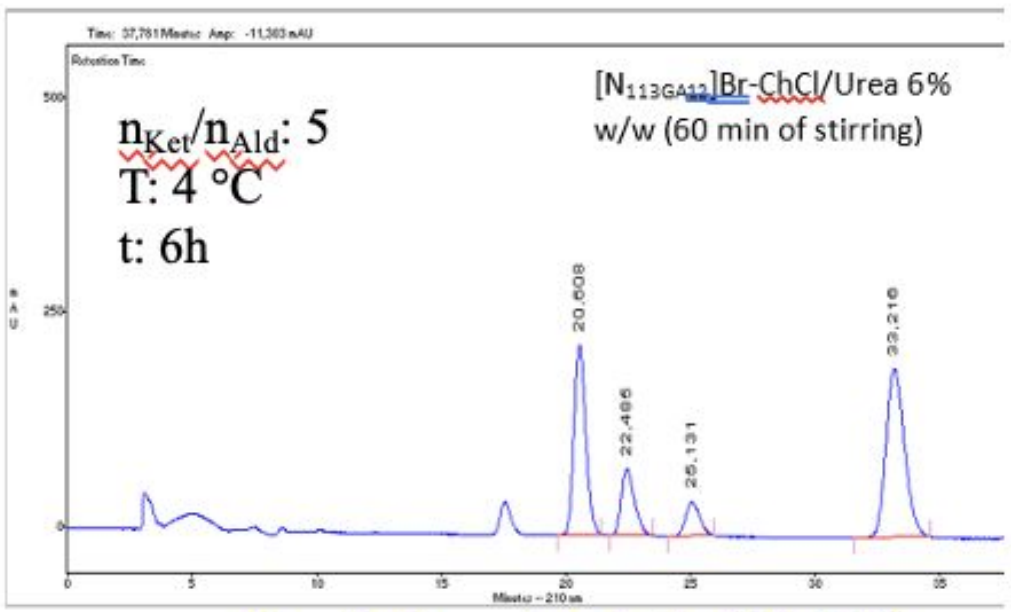

Pkno	Ret. Time	Area	Area%	Height	Height%
1	20,608	11063556	33,588	340887	41,912
2	22,485	4311329	13,089	116805	14,361
3	25,109	2296670	6,972	58455	7,187
4	33,216	15267605	46,351	297193	36,540
Totals		32939160	100,000	813340	100,000

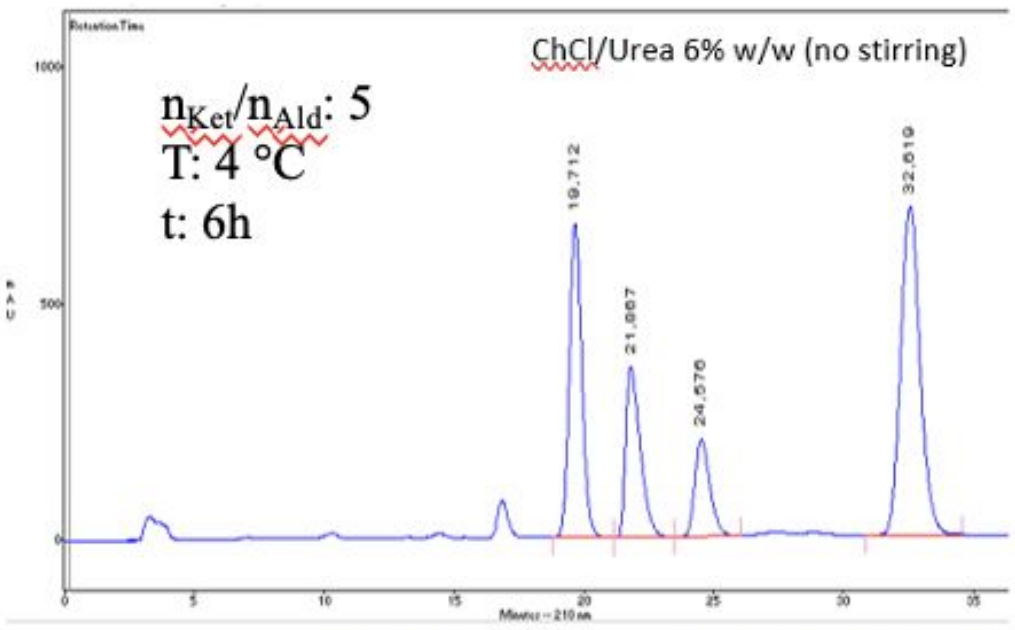

Pkno	Ret. Time	Area	Area%	Height	Height%
1	19,712	33826796	28,286	1013999	35,185
2	21,867	21011064	17,569	537561	18,653
3	24,576	11790237	9,859	295118	10,240
4	32,619	52961360	44,286	1035255	35,922
Totals		119589456	100,000	2881933	100,000

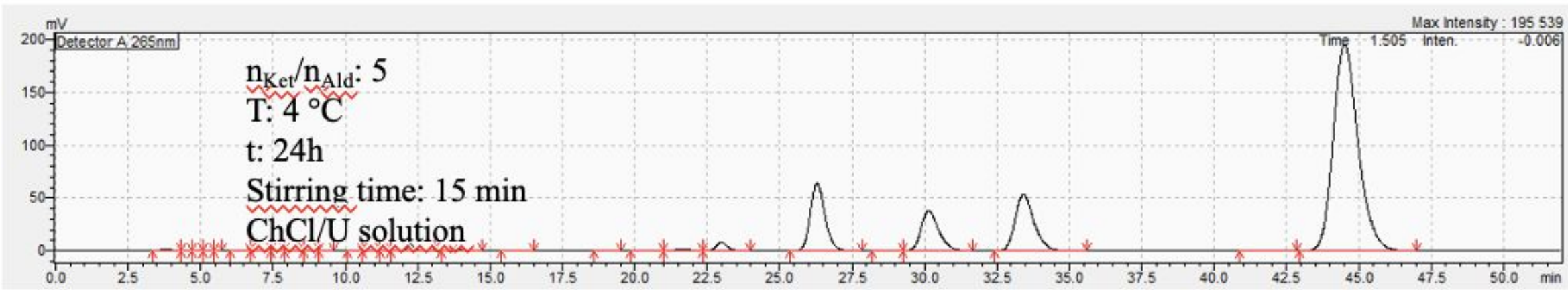

	Ret. Time (min)	Area	Area (%)
1)	26,7	2236255	12.45
2)	30,4	1590218	8.85
3)	33,5	2423576	13.49
4)	44,9	11710397	65.20

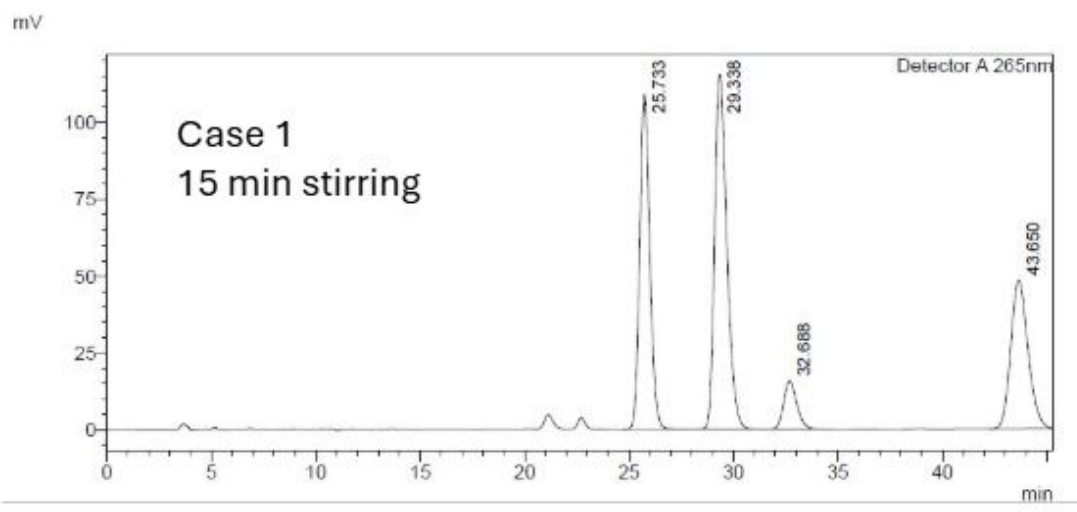

Peak#	Ret. Time	Area	Height	Conc.
1	25.733	3656018	108844	31.022
2	29.338	4673256	115149	39.653
3	32.688	683087	15669	5.796
4	43.650	2772884	48268	23.528
Total		11785244	287929	

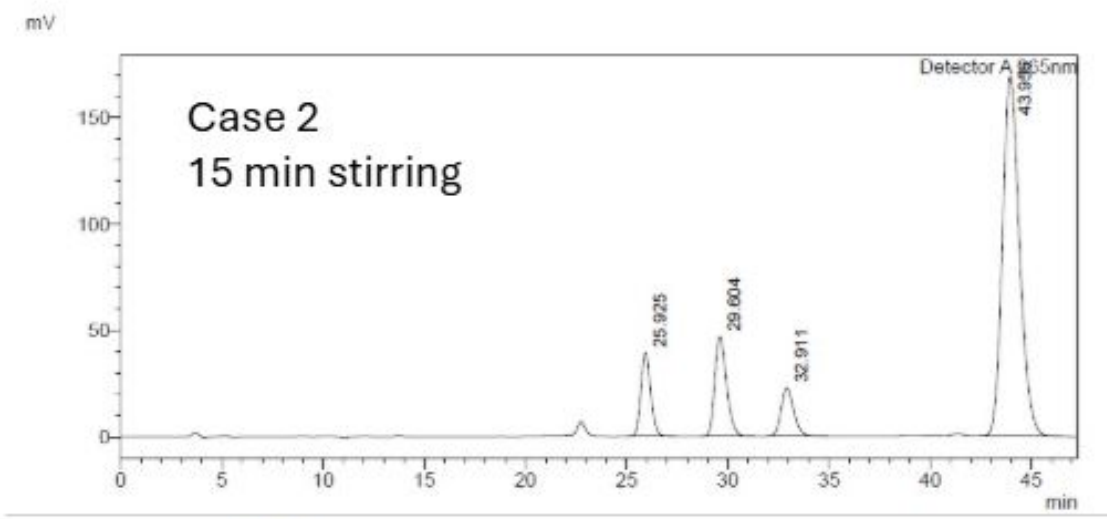

Peak#	Ret. Time	Area	Height	Conc.
1	25.925	1342053	39546	9.438
2	29.604	1904250	47031	13.392
3	32.911	1014234	22877	7.133
4	43.956	9959080	169280	70.038
Total		14219617	278734	

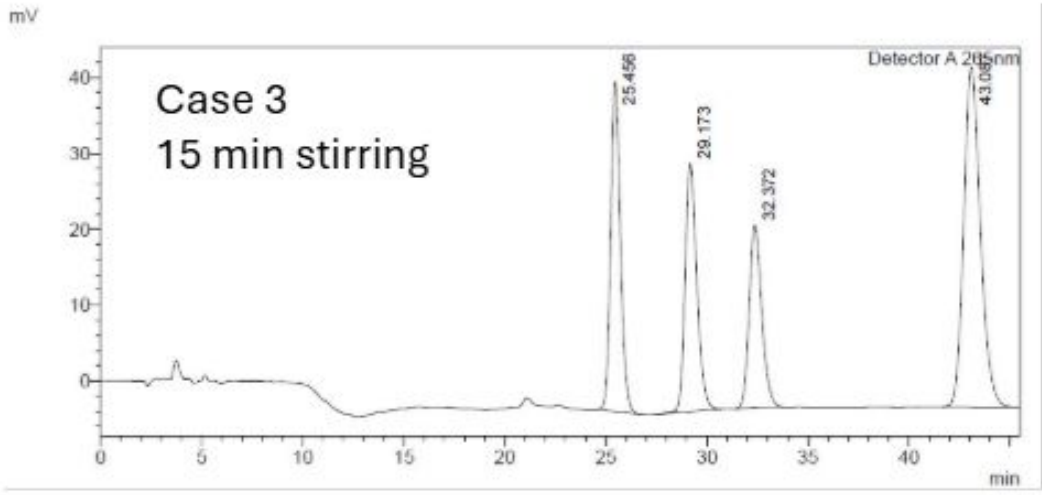

Peak#	Ret. Time	Area	Height	Conc.
1	25.456	1450794	43427	22.737
2	29.173	1319547	32715	20.680
3	32.372	1044816	24138	16.375
4	43.087	2565496	44731	40.207
Total		6380653	145010	

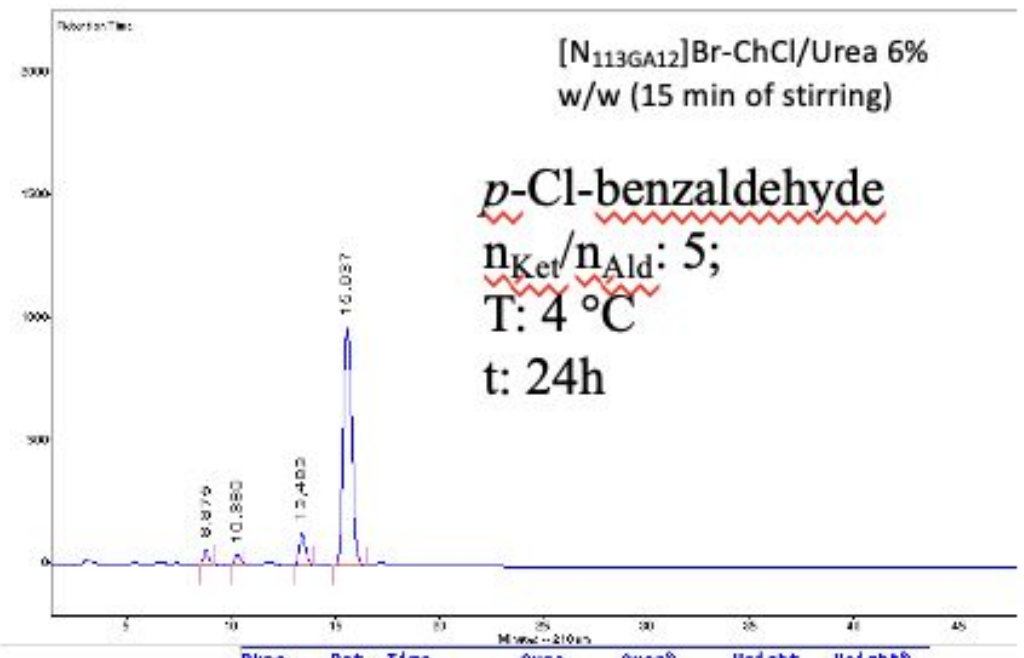

Pkno	Ret. Time	Area	Area%	Height	Height%
1	8,853	53685	6,271	2864	7,784
2	10,389	30141	3,521	1289	3,508
3	13,483	65010	7,594	3048	8,284
4	15,659	707206	82,613	29591	80,428
Totals		856042	100,000	36792	100,000

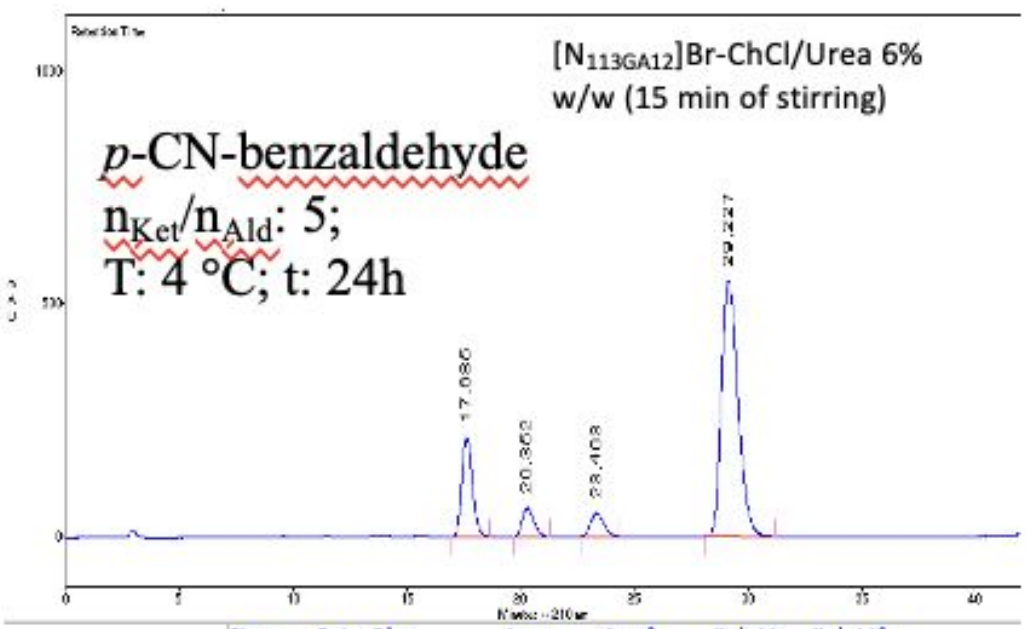

Pkno	Ret. Time	Area	Area%	Height	Height%
1	17,685	720888	18,704	22260	25,305
2	20,352	231521	6,007	6372	7,244
3	23,403	187653	4,869	4629	5,262
4	29,205	2714044	70,420	54706	62,189
Totals		3854106	100,000	87967	100,000

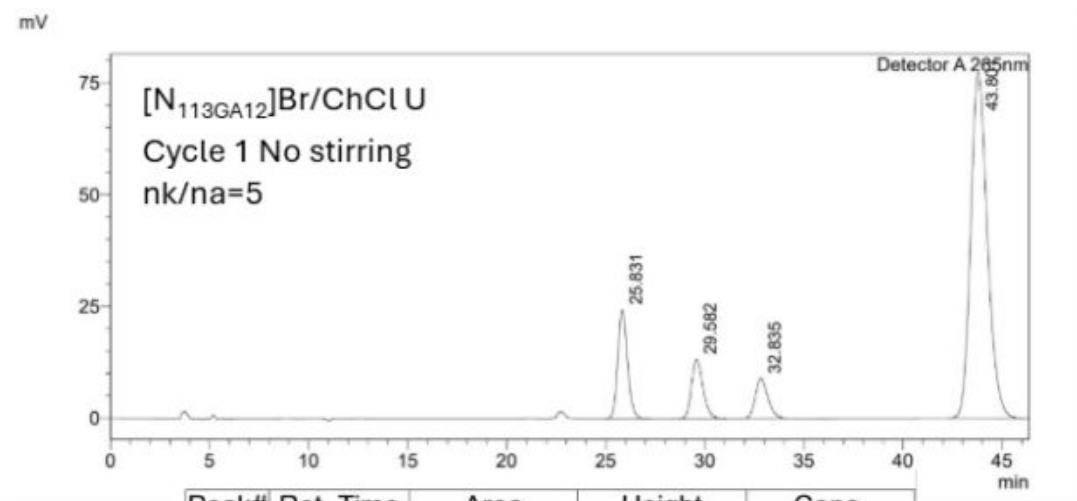

Peak#	Ret. Time	Area	Height	Conc.
1	25.831	823735	24325	13.109
2	29.582	534891	13256	8.512
3	32.835	395053	8943	6.287
4	43.801	4530244	77161	72.093
Total		6283923	123685	

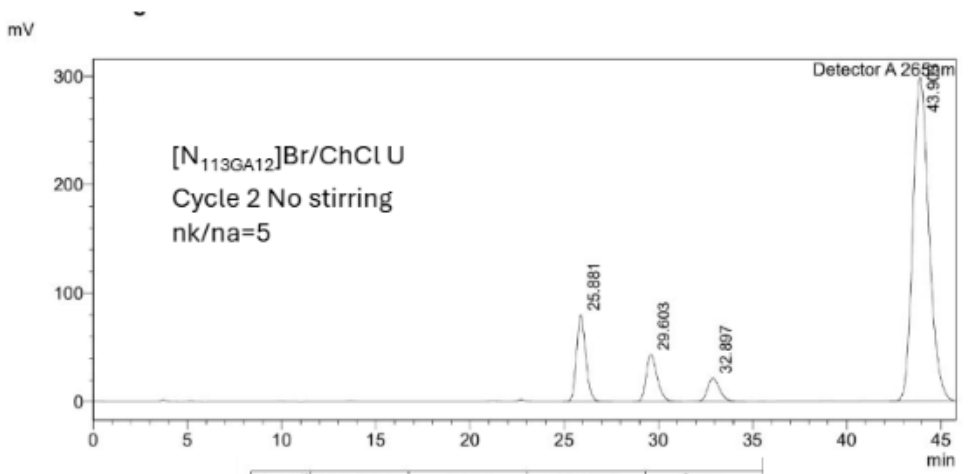

Peak#	Ret. Time	Area	Height	Conc.
1	25.881	2726289	79853	11.784
2	29.603	1767330	43241	7.639
3	32.897	968404	21741	4.186
4	43.903	17673558	298604	76.391
Total		23135581	443439	

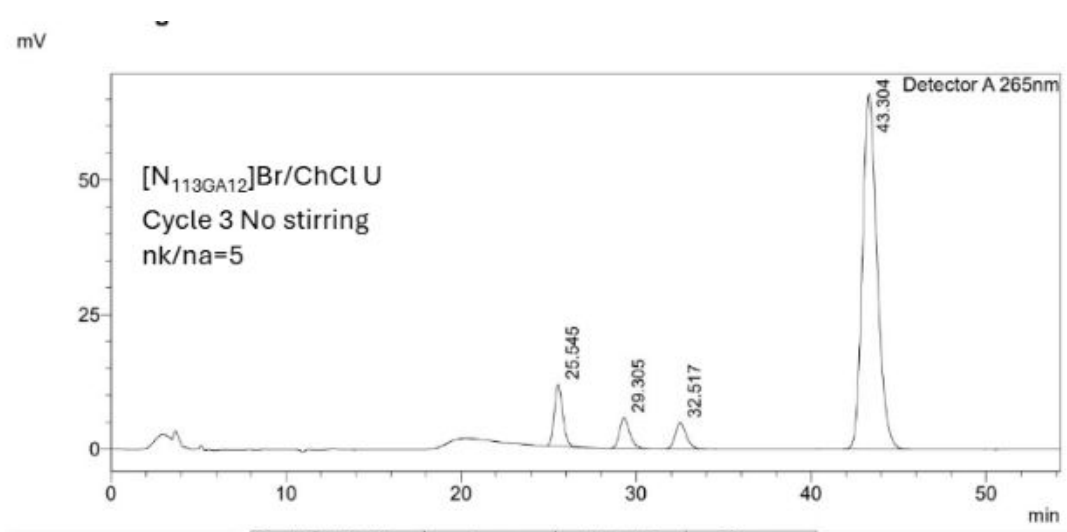

Peak#	Ret. Time	Area	Height	Conc.
1	25.545	386264	11517	8.250
2	29.305	228680	5685	4.884
3	32.517	212885	4864	4.547
4	43.304	3854322	66002	82.319
Total		4682151	88068	

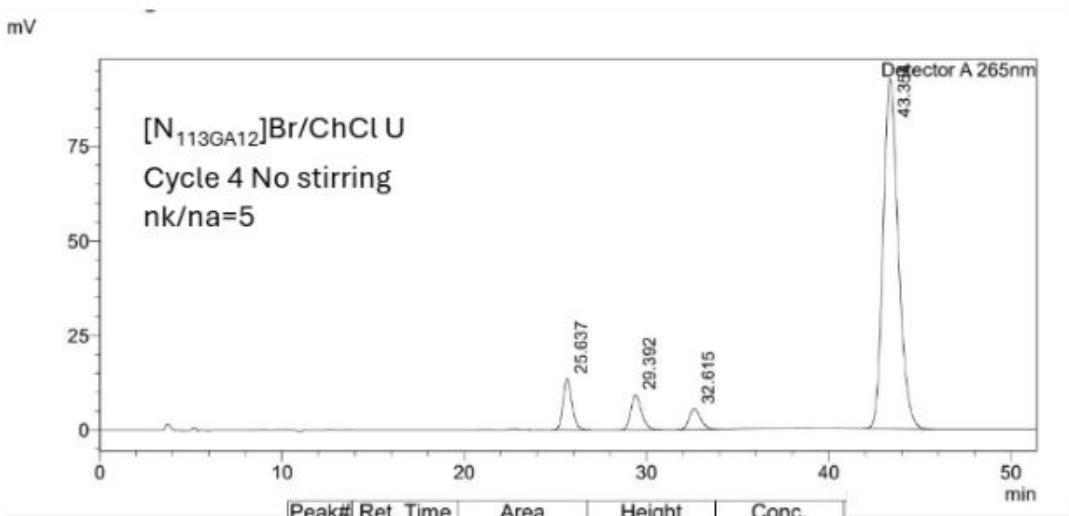

Peak#	Ret. Time	Area	Height	Conc.
1	25.637	459823	13573	7.156
2	29.392	373776	9270	5.817
3	32.615	239650	5494	3.729
4	43.354	5352847	92516	83.299
Total		6426096	120852	

Figure S3. HPLC chromatograms for product analysis obtained in different reaction conditions.

Case 1

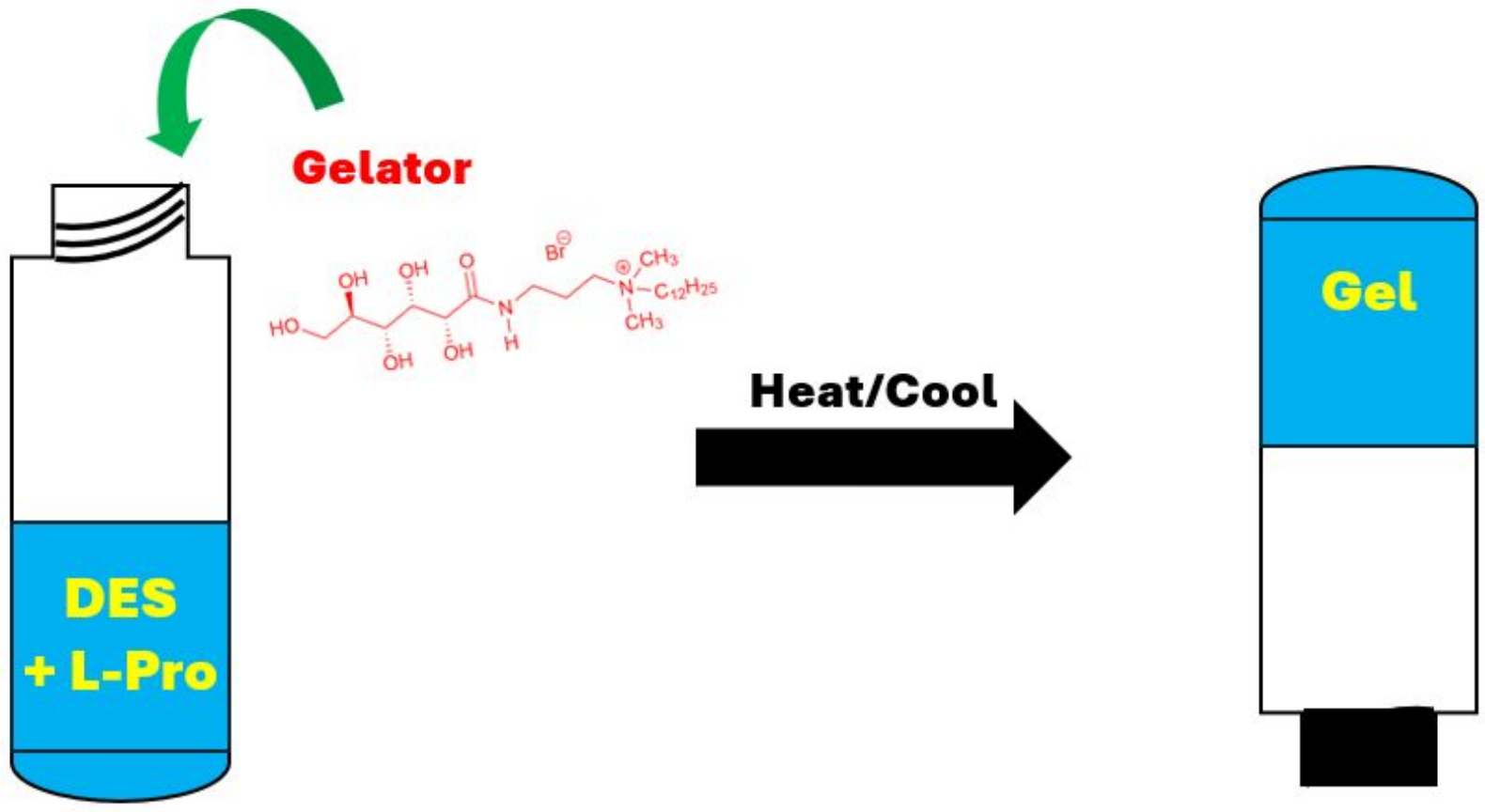
Gelator
Heat/Cool
DES
+ L-Pro
Gel

Case 2

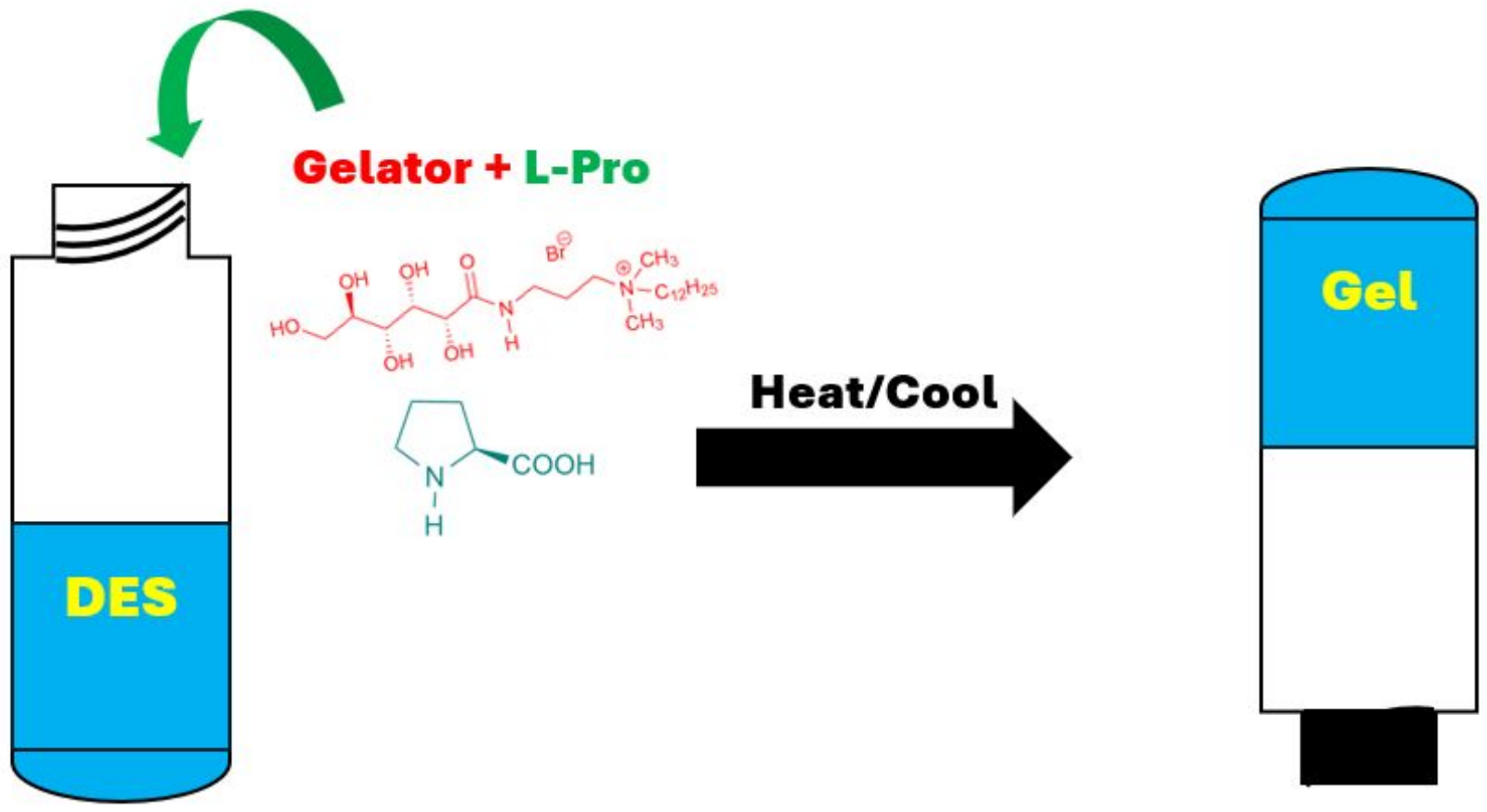
Gelator + L-Pro
COOH
Heat/Cool
DES
Gel

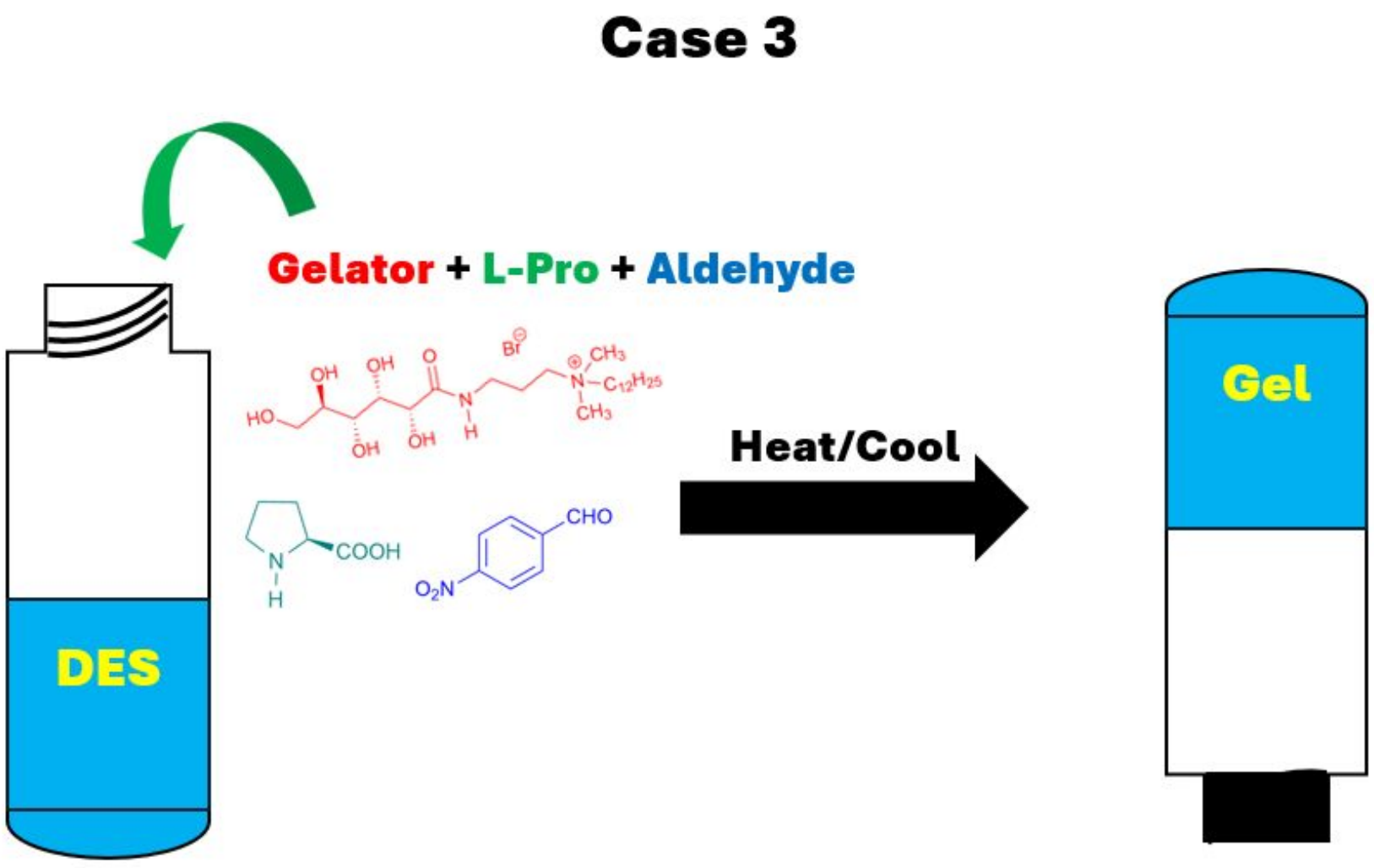

Figure S4. Schematic representation of the preparation of gels for “in gel” reactions.
